# Striatal Response to Reward Anticipation as a Biomarker for Schizophrenia and Negative Symptoms: Effects, Test-Retest Reliability, and Stability Across Sites

**DOI:** 10.1093/schbul/sbae046

**Published:** 2024-04-19

**Authors:** Fabien Carruzzo, Mariia Kaliuzhna, Noémie Kuenzi, Tal Geffen, Teresa Katthagen, Florian Schlagenhauf, Stefan Kaiser

**Affiliations:** Department of Psychiatry, Clinical and Experimental Psychopathology Laboratory, University Hospital Geneva, Thônex, Switzerland; Department of Psychiatry, Clinical and Experimental Psychopathology Laboratory, University Hospital Geneva, Thônex, Switzerland; Department of Psychiatry, Clinical and Experimental Psychopathology Laboratory, University Hospital Geneva, Thônex, Switzerland; Department of Psychiatry and Neurosciences, Charité—Universitätsmedizin Berlin, Berlin, Germany; Department of Psychiatry and Neurosciences, Charité—Universitätsmedizin Berlin, Berlin, Germany; Department of Psychiatry and Neurosciences, Charité—Universitätsmedizin Berlin, Berlin, Germany; Department of Psychiatry, Clinical and Experimental Psychopathology Laboratory, University Hospital Geneva, Thônex, Switzerland

**Keywords:** fMRI, ventral striatum, Monetary Incentive Delay task

## Abstract

**Background:**

Ventral striatal hypoactivation during reward anticipation has consistently been observed in patients with schizophrenia. In addition, that hypoactivation has been shown to correlate negatively with negative symptoms, and in particular with apathy. However, little is known about the stability of these results over time and their reliability across different centers.

**Methods:**

In total, 67 patients with schizophrenia (15 females) and 55 healthy controls (13 females) were recruited in 2 centers in Switzerland and Germany. To assess the neural bases of reward anticipation, all participants performed a variant of the Monetary Incentive Delay task while undergoing event-related functional magnetic resonance imaging at baseline and after 3 months. Stability over time was measured using intra-class correlation (ICC(A,1)) and stability between centers was measured with mixed models.

**Results:**

Results showed the expected ventral striatal hypoactivation in patients compared to controls during reward anticipation. We showed that these results were stable across centers. The primary analysis did not reveal an effect of time. Test-retest reliability was moderate for controls, and poor for patients. We did not find an association between ventral striatal hypoactivation and negative symptoms in patients.

**Conclusions:**

Our results align with the hypothesis that ventral striatal activation is related to modulation of motivational saliency during reward anticipation. They also confirm that patients with schizophrenia show impaired reward anticipation. However, the poor test-retest reliability and the absence of an association with symptoms suggests that further research is needed before ventral striatal activity can be used as a biomarker on the individual patient level.

## Introduction

Reward anticipation is a component of motivation that evaluates future incentives’ value to optimize goal-directed actions. A standard and robust functional magnetic resonance imaging (fMRI) method to assess the neural bases of reward anticipation is the Monetary Incentive Delay task (MID).^1^ In this simple task, participants are presented with a cue that indicates the amount of reward they can win per trial. The neural correlates of reward anticipation during fMRI are then modeled by comparing BOLD response related to high reward cues compared to neutral cues (this approach is used in the present paper, however, depending on the version of the MID task, other modeling approaches have also been used). Previous fMRI studies using the MID with healthy controls have shown robust activations in the ventral and dorsal striatum, anterior cingulate cortex, anterior insula, and thalamus.^2,3^

Reduced ventral striatal activity during reward anticipation has been repeatedly shown in patients with schizophrenia,^4–6^ in offspring,^7,8^ and in first-degree relatives^9^ of patients with schizophrenia. It has therefore been proposed as a neural biomarker of schizophrenia.

In addition, ventral striatal activity in patients with schizophrenia is associated with negative symptoms^4,6^: patients who exhibit lower ventral striatal activity show higher negative symptom severity. Moreover, this link may be specific to apathy symptoms and not diminished expression,^10–13^ 2 dimensions of negative symptoms. This indicates that apathy and diminished expression could arise from different neural deficiencies, with a link between apathy and the reward system. Based on these results, ventral striatal activation has also been suggested as a neural biomarker for negative symptoms, which could be of high interest for detection of target engagement in clinical trials.

However, for a neural biomarker to be useful in clinical research, several criteria have to be met. First of all, the biomarker reflects the underlying process of interest. Deficits in ventral striatal activity have been robustly shown in schizophrenia, and there is accumulating evidence for a link with motivation impairments underlying negative symptoms. However, there are also some inconsistent findings.^14,15^ Second, to be fully utilized as a biomarker, ventral striatal activity still needs to show stability over time in patients and across centers. Of note, ventral striatal activity has been shown to be stable over time in healthy controls, with intra-class correlation (ICC) coefficients ranging from 0.68 to 0.73.^9^

The main goal of the present study was therefore to assess those characteristics of ventral striatal activation during reward anticipation which would be necessary for its future use as an imaging biomarker, eg, for measuring target engagement in clinical trials. To this end, using the MID task, we acquired data from a large cohort of patients with schizophrenia (*N*_SZ_ = 67) and healthy controls (*N*_HC_ = 55) in 2 different sites and at 2 different time points.

We had the following aims and hypotheses. First, we aimed to replicate and extend previous findings regarding neural differences between healthy controls and patients with schizophrenia during reward anticipation. We hypothesized that patients with schizophrenia would show lower activations in the ventral striatum compared to healthy controls. Second, we aimed to measure the biomarker at 2 timepoints to assess test-retest reliability. We hypothesized that ventral striatal activity would remain stable over time, because no specific intervention was provided. Third, we aimed to assess robustness of findings across centers. We expected that our results would be stable across sites. Fourth, we aimed to assess the association between ventral striatal activation and apathy. We expected a negative association between ventral striatal activation and apathy. We also hypothesized that this relationship would be stable over time.

## Methods and Materials

### Participants

We performed a power analysis, to have a sufficient number of patients for the correlations between reward anticipation brain response and symptoms. Given a probability of type I error of 5%, detecting an effect of this magnitude with 90% probability requires at least 46 participants in a group. Dropouts in our previous cross-sectional fMRI studies have been 10% or less. To account for the potentially increased dropout due to the longitudinal design, we assume a dropout rate of 30%. Thus, the power calculations result in a sample size of 66 participants at inclusion in each center which would allow to calculate separate correlations at each site. Due to the SARS-Cov2 pandemic, these numbers were unfortunately not reached. However, across centers, we have sufficient power to observe correlations were they indeed present.

Patients with schizophrenia (SZ) were recruited from outpatient units of the University Hospital of Geneva, Switzerland (Switzerland; *n*_SZ_Switzerland_ = 50) and from inpatient and outpatient units of the Charité University Hospital of Berlin, Germany (Germany; *n*_SZ_Germany_ = 36, *n*_SZ_TOTAL_ = 86). All patients with schizophrenia were stable, both symptom- and medication-wise. Healthy controls (HC) were recruited at session 1 from the general population in Geneva (*n*_HC_Switzerland_ = 31) and Berlin *(n*_HC_Germany_ = 35, *n*_HC_TOTAL_ = 66). HC were matched as best as possible to the age, gender, and parents’ education of the patient group.

Nineteen patients with SZ and 11 HC dropped out between sessions 1 and 2. The final analyses were performed on data from 67 patients with SZ (*n*_SZ_Germany_ = 28, *n*_SZ_Switzerland_ = 39) and 55 HC (*n*_HC_Germany_ = 26, *n*_HC_Switzerland_ = 29) who took part in both sessions. The study was approved by the local ethics committees in Geneva and Berlin. All participants provided written informed consent.

All participants were screened using the Mini-International Neuropsychiatric Interview (MINI)^16^ for DSM-IV to confirm clinical diagnosis and to exclude participants with Axis I disorder comorbidities. Other exclusion criteria for patients included the presence of florid psychotic symptoms (ie, values higher than 4 on the Positive and Negative Syndrome Scale (PANSS) positive subscales) and extrapyramidal side effects. Exclusion criteria for healthy controls comprised the presence of any psychotic symptom and the presence of a psychiatric diagnostic in the immediate family. Finally, all participants were required to have a sufficient level of French or German to take part in the study.

### Clinical and Cognitive Assessment

All participants underwent a complete clinical assessment in French in Switzerland and in German in Germany. Negative symptom severity was primarily assessed using the Brief Negative Symptom Scale (BNSS).^17,18^ Apathy and diminished expression dimensions were extracted as defined in previous studies.^19,20^ Specifically, the apathy dimension was composed of the sums of avolition, asociality, and anhedonia scores, while diminished expression included alogia and flat affect scores. Raters at both sites were trained by SK who was the principal investigator of the validation study of the German version of the BNSS.^19^

Positive and negative symptoms were assessed using the Positive and Negative Syndrome Scale,^21^ and scores were defined based on the factor decomposition from Wallwork, Fortgang.^22^ Depression symptoms were evaluated with the Calgary Depression Scale (CDS).^23^ Extrapyramidal symptoms were evaluated with the St. Hans Rating Scale (SHRS).^24^ Cognition was assessed using the Brief Assessment of Cognition in Schizophrenia (BACS).^25^ Due to the variety of antipsychotics prescribed to patients, we calculated risperidone equivalence based on the defined daily doses method.^26^

### Experimental Task

We assessed reward anticipation using a modified version of the MID task (Knutson et al^1^) developed by Simon, Cordeiro^27^ and reprogrammed with the Psychophysics Toolbox MATLAB extensions.^28–30^ Every trial started with a centered cue indicating the maximum amount of reward the participant could win within that trial (0CHF, 0.40CHF, 2CHF in Switzerland, and 0EUR, 0.20EUR, and 1EUR in Germany, to match the socio-economic context of each country) presented for 0.75 s, followed by a delay of 2.5–3 s. The participants were then presented with 3 circles and had to identify the outlier circle as fast as possible. This target screen lasted until a button press, or for a maximum of 1 s. Participants then saw a feedback screen for 2 s. Trials with no response or with a wrong response were considered errors. In case of a correct answer, the feedback screen indicated the amount of reward won in that trial. The amount of reward was calculated on a trial basis as a percentage of the maximum amount, based on the mean response time of the previous 15 trials. Through this procedure similar reward amounts were received by all participants. Each trial concluded with a jittered intertrial interval screen (ITI, 1–9 s, *m*_ITI_ = 3.5 s). Participants performed 1 trial run (12 trials) outside the scanner, to get acquainted with the task, then 1 trial run (6 trials) inside the scanner to get used to performing while lying in the machine and using response boxes. Then, they performed 2 test runs (36 trials each) in the scanner. The test runs lasted about 6 min each. Participants were informed at the beginning of the task that they would receive the total amount of reward won during the 2 test runs. At the end of the experiment participants effectively received the amount won, along with the reimbursement for their participation.

### Experimental Design

This longitudinal study consisted of 2 sessions. The term session refers to the 2 groups of visits separated by a waiting period of 90 ± 14 days. Due to the SARS-Cov2 pandemic, several session 2 appointments had to be postponed. In the end, the average duration between our 2 session was of 106.87 days (*SD*_days_ = 22.51). Each session was further split into 2 visits, separated by a maximum of 7 days. During the first visit of session 1, participants underwent detailed demographic, clinical, and cognitive interviews. The MINI was also used during the first visit of session 1 to assess the presence of comorbid disorders. The first visit lasted between 2 and 5 h and could be split if patients experienced high fatiguability. The second visit was dedicated to MRI acquisitions, with both MID and structural data acquired sequentially. The second visit lasted about 2 h in total. The 2 visits of session 2 followed the same procedure, without the MINI.

### Clinical, Cognitive, and Behavioral Data Analyses

Clinical, cognitive, and behavioral statistical analyses were performed using R.^31^ Main demographic data were acquired only at session 1. Analyses on age and gender were calculated using a general linear model with Group and Site as between-subject factors. Analyses on participant-, father-, and mother-education level were performed using a mixed model with Group and Site as between-subject factors and participants as a random effect. *T*-tests and Wilcoxon tests were used to compare SZ scores between Switzerland and Germany, and HC scores between Switzerland and Germany, for sessions 1 and 2 separately. ICC(A,1) were calculated on SZ and HC scores to assess stability of clinical and cognitive variables between the 2 sessions.

We modeled response time for the correct MID trials as the time in milliseconds between the presentation of the target and button press. We defined 3 mixed models on response time, accuracy, and total reward amount, with Group (SZ vs HC), Site (Switzerland vs Germany) as the between-subject factors and Reward (High Reward vs No Reward) and Session (session 1 vs session 2) as the within-subject factors. Participants were introduced in the mixed models as a random effect. The total reward amount was doubled for Germany, to match amounts received in Switzerland due to the economic differences in the 2 countries. ICC were calculated on SZ and HC scores to assess stability of the behavioral results between the 2 sessions.

### MRI Acquisitions

Imaging data were acquired on a Siemens Magnetom Prisma 3.0T whole-body scanner at Campus Biotech in Geneva and on a Siemens Magnetom Prisma Fit 3.0T whole-body scanner at Charité Hospital in Berlin. Both MRI machines were equipped with a 64-channel head coil. Functional runs had between 359 and 386 images using an echo-planar image (EPI) sequence with 66 slices acquired in an interleaved fashion, with a multiband acceleration factor of 6. The in-plane resolution was 2 × 2 mm, 2 mm slice thickness, and a field of view of 224 mm. Volume acquisition had a TR of 1000 ms, a TE of 32 ms, and a flip angle of 50°. Anatomical data were acquired using an MPRAGE sequence in 208 sagittal plane slices of 256 × 256 mm with a 1 × 1 mm resolution and a slice thickness of 1 mm.

### Image Preprocessing

We detected motion and susceptibility artifacts using the Art toolbox (http://web.mit.edu/swg/software.htm), defining outliers as scans with head motion above 2 mm and/or changes in mean signal intensity above 9. In total, 1.04% of all scans were flagged as outliers. The highest percentage of outlier in 1 single participant was 15%. All outlier scans were scrubbed from subsequent analyses. No participant was excluded based on these analyses (to assess whether motion still affected our results, we performed a set of analyses using framewise displacement, reported in supplementary analyses. We find no effects of motion).

Results included in this manuscript come from preprocessing performed using the latest version of FMRIPREP,^32^ a Nipype-based tool. Each T1w (T1-weighted) volume was corrected for INU (intensity nonuniformity) using N4BiasFieldCorrection v2.1.0^33^ and skull-stripped using antsBrainExtraction.sh v2.1.0 (using the OASIS template). Spatial normalization to the ICBM 152 Nonlinear Asymmetrical template version 2009c^34^ was performed through nonlinear registration with the antsRegistration tool of ANTs v2.1.0,^35^ using brain-extracted versions of both T1w volume and template. Brain tissue segmentation of cerebrospinal fluid (CSF), white matter (WM), and gray matter (GM) was performed on the brain-extracted T1w using fast (FSL v5.0.9).^36^

Functional data was slice time corrected using 3dTshift from AFNI v16.2.07^37^ and motion corrected using mcflirt (FSL v5.0.9).^38^ This was followed by co-registration to the corresponding T1w using boundary-based registration^39^ with 6 degrees of freedom, using flirt (FSL). Motion correcting transformations, BOLD-to-T1w transformation, and T1w-to-template (MNI) warp were concatenated and applied in a single step using antsApplyTransforms (ANTs v2.1.0) using Lanczos interpolation. Many internal operations of FMRIPREP use Nilearn,^40^ principally within the BOLD-processing workflow. Finally, functional images were smoothed on SPM12 using a 5 mm full-width at half-maximum Gaussian kernel.

### First-Level Statistics

First and second-level fMRI analyses were performed in MATLAB R2022a (Mathworks, Natick) using SPM12 (Statistical Parametric Mapping, Welcome Trust Centre for Neuroimaging, London, UK). We defined our event-related design using a general linear model (GLM). We included 3 anticipation regressors (no reward, low reward, and high reward), and 3 consumption regressors (ie, at the level of the feedback stage) for the same conditions. We parametrically modulated the low and high reward consumption regressors with the amount of reward received per trial, thus adding 2 regressors to the model. An additional regressor modeled target presentation. Three error regressors modeling error in the anticipation, consumption, and target phases were added in case participants made errors in that session (in which case error trials were removed from the main regressors). Finally, we added outlier scans defined by Art toolbox in single columns (ie, to be scrubbed from subsequent analyses), 6 movement parameters, and framewise displacement as covariates of no interest. In total, the GLM comprised 12 regressors that were convolved using the canonical hemodynamic response function. We created a first-level contrast for reward anticipation by subtracting the no reward condition from the high reward condition [high reward > no reward].

### Categorical and Covariate Second Level Analyses

Second-level analyses on reward anticipation integrated individual contrast images defined during the first-level analyses. Group comparisons were performed using 2-sample *t*-tests, while covariate analyses were performed with 1-sample *t*-tests.

#### Striatum Regions of Interest Analyses.

Masks for region of interest (ROI) analyses were taken from Mawlawi, Martinez.^41^ Six regions were defined: the left ventral striatum (lVS) and the right ventral striatum (rVS). We also performed complementary analyses on the left and right dorsal striatum (lDS and rDS) and the left and right posterior putamen (lpPut and rpPut). ROI activity was extracted from each ROI using marsbar. ROI analyses were then performed in R. We defined 6 mixed-effects models of ROI activity with Group (SZ vs HC), Site (Switzerland vs Germany) as between-subject factors and Session (session 1 vs session 2) as a within-subject factor and participants as random effect.

#### Dimensional Relationships.

To evaluate any associations between the reward anticipation brain response and clinical variables within the patient group, we performed mixed-effects models of ROI activity looking at the correlations with negative symptoms (ie, BNSS apathy, diminished expression, and total negative symptoms), positive symptoms (ie, PANSS positive factor), depressive symptoms (ie, Calgary total score), and cognition (ie, BACS total score), with Site (Switzerland vs Germany) as between-subject factor and Session (session 1 vs session 2) as a within-subject factor and participants as random effect. Similarly, mixed-effects models were used to look at correlations between ROIs activity and response time speeding (ie, the difference between the mean response time for high reward trials and for low reward trials), accuracy, and total reward amount. For session 1 all patients (*N* = 86) were included in the model to maximize power.

## Results

### Sample Characteristics

Demographic and clinical characteristics of our samples can be found in table 1. All groups were aged- and gender-matched. Participants education was not matched anymore between our samples as SZ were less educated than HC. However, their parents’ education was still matched.

**Table 1.** Demographic and Clinical Characteristics of Patients With Schizophrenia and Healthy Controls at Session 1

	Session 1							
	Berlin		Geneva		All		Statistics	
	SZ (*N* = 28)	HC (*N* = 26)	SZ (*N* = 39)	HC (*N* = 29)	SZ (*N* = 67)	HC (*N* = 55)		
Demographics								
Age (years)	38.0 (1.95)	37.7 (1.49)	38.6 (1.58)	38.8 (1.38)	38.4 (1.22)	38.3 (1.01)		
Gender	0.82 (0.07)	0.73 (0.09)	0.74 (0.07)	0.79 (0.08)	0.78 (0.05)	0.76 (0.06)		
Education (years)								
Participant	13.0 (0.59)	14.8 (0.51)	12.6 (0.47)	14.8 (0.30)	12.8 (0.37)	14.8 (0.28)	Group (*F* = 16.83, ***)	
Father	13.1 (0.61)	13.2 (0.46)	11.3 (0.78)	12.6 (0.62)	12.1 (0.52)	12.9 (0.39)		
Mother	12.8 (0.70)	13.1 (0.45)	10.8 (0.66)	12.1 (0.61)	11.6 (0.50)	12.6 (0.39)	Site (*F* = 5.59, *)	
Illness								
Age of onset	24.28 (1.90)		24.58 (1.05)		24.45 (0.83)			
Duration	13.71 (1.90)		13.63 (1.30)		13.67 (1.09)			
							CH vs GE	
Clinical variables							SZ	HC
BNSS								
Apathy	16.9 (1.89)	2.19 (0.67)	12.8 (1.31)	0.52 (0.16)	14.6 (1.12)	1.31 (0.34)		
Diminished expression	8.61 (1.36)	0.92 (0.39)	7.26 (1.11)	0.62 (0.30)	7.82 (0.86)	0.76 (0.24)		
Total	25.5 (2.99)	3.12 (0.88)	20.1 (2.19)	1.14 (0.33)	22.4 (1.81)	2.07 (0.47)		
PANSS								
Negative factor	15.7 (1.32)	6.96 (0.35)	13.7 (0.94)	6.28 (0.15)	14.5 (0.78)	6.60 (0.19)		*W* = 470, *
Positive factor	7.43 (0.67)	4.27 (0.14)	6.49 (0.39)	4.10 (0.06)	6.88 (0.36)	4.18 (0.07)		
Total	57.7 (3.87)	33.2 (0.85)	48.1 (1.57)	31.3 (0.37)	52.1 (1.93)	32.2 (0.46)		
CDS total	3.71 (0.89)	0.85 (0.28)	2.64 (0.48)	0.34 (0.14)	3.09 (0.47)	0.58 (0.15)		
BACS total (*z* score)	−1.33 (0.26)	1.17 (0.84)	−1.63 (0.16)	0.64 (0.19)	−1.50 (0.15)	0.89 (0.40)		
SHRS parkinsonism score	1.61 (0.70)		3.10 (0.65)		2.48 (0.49)		*W* = 290.5, ***	
RIS equivalence	5.52 (0.86)		4.85 (0.45)		5.13 (0.44)			

Scores displayed as Mean (SE). ***P < .001, **P < .01, *P < .05.

In terms of symptoms, at session 1, SZ were matched between Switzerland and Germany, except for the SHRS parkinsonism score (*W* = 290.5, *P* < .001). HC characteristics were similar between Switzerland and Germany on most variables, except for the PANSS negative factor (*W* = 470, *P* < .05). Similarly, SZ and HC at session 2 were matched on most clinical variables, with a few more differences. Group differences at session 2 can be found in supplementary table 2. Stability of clinical scores between sessions 1 and 2 can be found in supplementary table 3.

### Behavioral Analyses

**Fig. 1.**
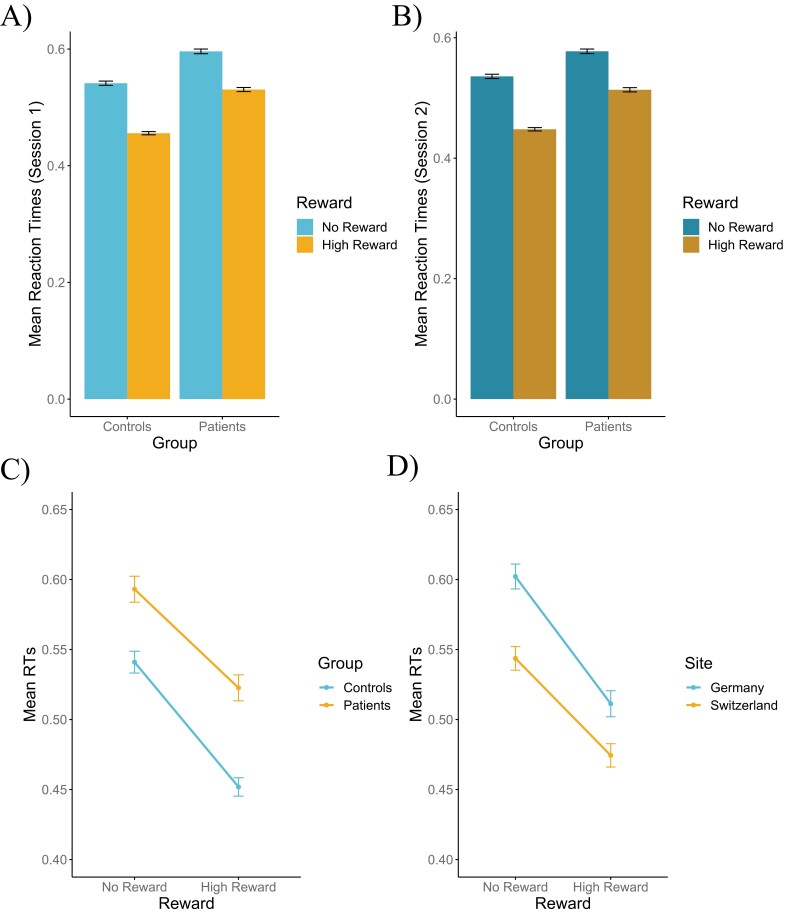
(A) Mean response times of high and no reward trials in patients with schizophrenia vs healthy controls in session 1. (B) Mean response times of high and no reward trials in patients with schizophrenia vs healthy controls in session 2. (C) Main effect of Group and Interaction effect between reward and group showing a greater speeding effect of high reward in healthy controls. (D) Main effect of Site and Interaction effect between Reward and Site, with a greater speeding effect of high reward in Germany compared to Switzerland.

The mixed model on response time (figure 1) indicated a main effect of Reward, where high reward trials yielded faster responses (*M*_RT_high_ = 0.49, *SD*_RT_high_ = 0.10) than no reward trials (*M*_RT_no_ = 0.57, *SD*_RT_no_ = 0.10; *F*(365.11) = 424.39, *P* < .001, η_p_^2^ = 0.54, CI_95%_ = [0.48, 1]). We also found a main effect of Group, where HC responded faster (*M*_RT_HC_ = 0.50, *SD*_RT_HC_ = 0.09) than SZ (*M*_RT_SZ_ = 0.56, *SD*_RT_SZ_ = 0.11; *F*(123.16) = 19.31, *P* < .001, η_p_^2^ = 0.14, CI_95%_ = [0.06, 1]), a main effect of Site, where participants in Switzerland responded faster than participants in Germany (*F*(123.06) = 12.20, *P* < .001, η_p_^2^ = 0.09, CI_95%_ = [0.03, 1]), and a main effect of Session, where participants responded slower in session 1 than in session 2 (*F*(365.85) = 9.88, *P* < .01, η_p_^2^ = 0.03, CI_95%_ = [0, 1]). The mixed model on response time also identified interaction effects. The first one was between Reward and Group (*F*(365.11) = 5.19, *P* < .05, η_p_^2^ = 0.01, CI_95%_ = [0, 1]), where HC showed greater reward-related speeding than SZ. The second one was between Reward and Site (*F*(365.11) = 7.12, *P* < .01, η_p_^2^ = 0.02, CI_95%_ = [0, 1]), where participants in Germany showed greater reward-related speeding than participants in Switzerland. The third one was between Site and Session (*F*(365.78) = 5.82, *P* < .05, η_p_^2^ = 0.02, CI_95%_ = [0, 1]), where participants in Germany had faster response times in session 2 than session 1, while the response times were the same in Switzerland. The last one was between Group, Site, and Session (*F*(365.91) = 8.25, *P* < .01, η_p_^2^ = 0.02, CI_95%_ = [0, 1]), indicating that the decrease in response time between the 2 sessions in Germany was driven by a decrease in response time in patients, mainly, while the response times of healthy controls were stable. No other effects reached significance (all *F*s < 1.9, all *P*s > .14). Additionally, response times showed good reliability across sessions 1 and 2 for SZ (*ICC*_RT_SZ_ = 0.86, *P* < .05) and for HC (*ICC*_RT_HC_ = 0.77, *P* < .05).

The mixed model on accuracy (supplementary figure 1A) showed no main effect of either Group, Site, or Session (*Fs* < 2.4, *P*s > .1). However, it identified an interaction effect between Group and Session (*F*(122) = 4.03, *P* < .05, η_p_^2^ = 0.03, CI_95%_ = [0, 1]), where HC had higher accuracy than SZ in session 1, but not in session 2. There were no other interaction effects (*Fs* < 0.7, *P*s > .4). Accuracy showed moderate reliability across sessions 1 and 2 for SZ (*ICC*_ACC_SZ_ = 0.56, *P* < .05) and poor reliability for HC (*ICC*_ACC_HC_ = 0.45, *P* < .05).

The mixed model on total reward amount (supplementary figure 1B) showed a significant effect of Group, where HC received higher amounts of reward (*M*_TRA_HC_ = 34.16, *SE*_TRA_HC_ = 0.47) than SZ (*M*_TRA_SZ_ = 32.44, *SE*_TRA_SZ_ = 0.43; *F*(122) = 6.90, *P* < .01, η_p_^2^ = 0.05, CI_95%_ = [0.01, 1]). There was no difference between sites and sessions, nor any interactions (all *F*s < 0.97, all *P* > .37). Total reward amounts showed poor reliability across sessions 1 and 2 for SZ (*ICC*_TRA_SZ_ = 0.37, *P* < .05) and for HC (*ICC*_TRA_HC_ = 0.36, *P* < .05).

### fMRI Analyses

#### Group Differences

**Fig. 2.**
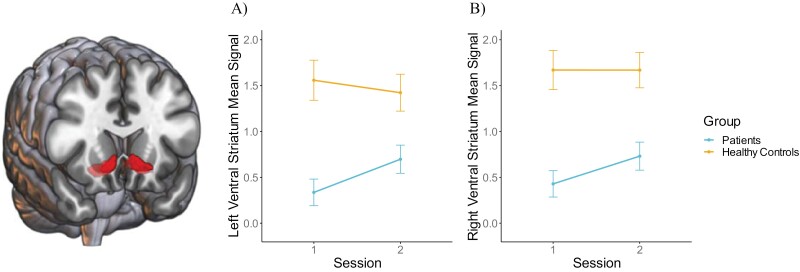
Main effect of Group in left (A) and right (B) ventral striatal mean signal showing greater activity in healthy controls compared to patients with schizophrenia. A trending interaction effect shows that patients with schizophrenia have more activity in session 2 than session 1.

. Regarding our primary hypothesis for VS ROIs, mixed models (figure 2) showed a significant effect of Group with HC showing increased BOLD response compared to SZ in lVS (*M*_lVS_HC_ = 1.49, *SE*_lVS_HC_ = 0.16; *M*_lVS_SZ_ = 0.55, *SE*_lVS_SZ_ = 0.14; *F*(122) = 20.62, *P* < .001, η_p_^2^ = 0.14, CI_95%_ = [0.06, 1]) and rVS (*M*_rVS_HC_ = 1.67, *SE*_rVS_HC_ = 0.16; *M*_rVS_SZ_ = 0.61, *SE*_rVS_SZ_ = 0.15; *F*(122) = 24.86, *P* < .001, η_p_^2^ = 0.17, CI_95%_ = [0.08, 1]). There was no effect of Session, nor any interaction (all *Fs* < 3.6, all *P*s > .06).

Analyses on DS and pPut activation can be found in supplementary analyses. Results indicated that these 2 ROIs have similar activation patterns as the VS.

Exploratory, whole-brain analyses (supplementary table 4) at session 1 showed categorical differences between HC and SZ, with HC having higher activations in the thalamus, right putamen/dorsal striatum/ventral striatum/amygdala, left putamen/dorsal striatum/ventral striatum/amygdala/thalamus, right anterior cingulate cortex, and left calcarine gyrus. SZ showed no higher activity compared to HC.

#### Stability Across Centers.

Stability of ventral striatal activation across centers was assessed in the mixed models described above. Our analyses showed no effect of Site on lVS (main effect *F*(122) = 0.7, *P* = .4; interaction with Group *F*(122) = 1.9, *P* = .2; interaction with Group and Session *F*(122) = 0.0001, *P* = .99) or rVS activity (main effect *F*(122) = 0.96, *P* = .3; interaction with Group *F*(122) = 0.6, *P* = .5; interaction with Group and Session *F*(122) = 0.5, *P* = .5). Finally, supplementary analyses show a strong spatial activation overlap between the 2 Sites (section 7.6). Ventral striatal activity can therefore be considered stable across centers. Mean and standard deviation values per center can be found in supplementary table 5.

#### Reliability of VS Activity Over Time.

Mixed models including Session did not show a significant difference between activity in sessions 1 and 2 suggesting that changes over time were limited on the group level.

We then calculated ICC coefficients to assess test-retest reliability. ICC analyses on ventral striatal activity showed poor reliability across sessions 1 and 2 for the lVS and rVS for SZ (*ICC*_lVS_SZ_ = 0.36, *P* < .05; *ICC*_rVS_SZ_ = 0.48, *P* < .05) and moderate reliability for HC (*ICC*_lVS_HC_ = 0.56, *P* < .05; *ICC*_rVS_HC_ = 0.65, *P* < .05).

Whole-brain analyses at session 2 using the [HC > SZ] results of session 1 as mask (voxel-level threshold of *P* = .05 FWE, cluster-level threshold of *P* = .05 FWE) showed similar higher activations in HC compared to SZ, but only in the right putamen/dorsal striatum/ventral striatum/amygdala and left putamen/dorsal striatum/ventral striatum/amygdala/thalamus clusters. Once again, SZ showed no higher activity compared to HC.

#### Dimensional Relationships With Symptoms

**Fig. 3.**
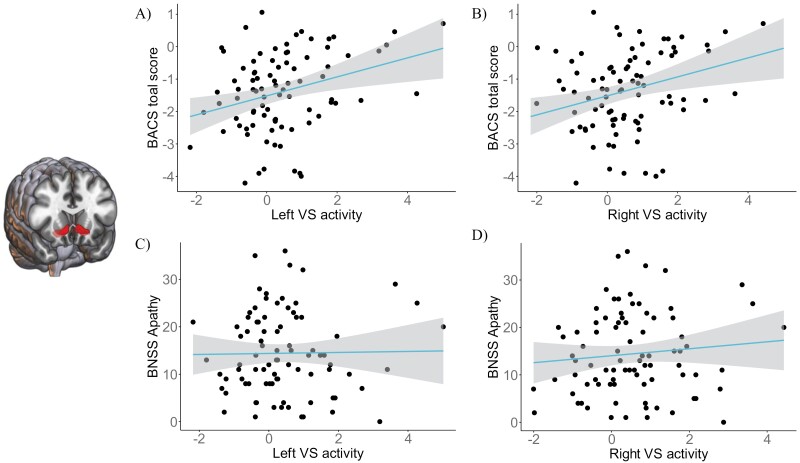
Increased cognitive scores (BACS) associated with higher left (A) and right (B) ventral striatal mean signal but no association between left (C) and right (D) ventral striatal mean signal and BNSS apathy scores in the complete sample of patients (*N* = 86).

. Contrary to our hypothesis we found no correlations between VS ROI activity in SZ and BNSS apathy (figure 3). The mixed-effects model did not show any correlation between VS activity and the symptom variables BNSS diminished expression, PANSS positive factor, Calgary Depression Scale total score (all *P*s > .05). Thus, there was no association between the VS ROI activity and these symptoms in either session 1 or session 2. In the full sample of 86 patients at session 1 the correlations with reward anticipation were as follows (uncorrected): apathy (lVS ρ < −0.001, *P* = .99; rVS ρ = 0.085, *P* = .44), diminished expression (lVS ρ = −0.07, *P* = .5; rVS ρ = −0.004, *P* = .97), positive symptoms (lVS ρ = −0.079, *P* = .47; rVS ρ = 0.07, *P* = .52), and depression (lVS ρ = 0.25, *P* = .023; rVS ρ = 0.26, *P* = .015).

However, the model did show a main effect of the BACS total score (ρ_lVS_ = 0.34, *P* = .0006; ρ_rVS_ = 0.3, *P* = .0036). Thus, while controlling for other symptoms, and independently of Session or Study site, patients showed a positive correlation between the activity in the left and right VS and cognition (figure 3). Those showing higher cognitive scores also showed more intact reward anticipation (this correlation is present for session 1 in the full sample of 86 patients, and in session 2 for the reduced sample of 67 (lVS ρ = 0.44, *P* = .0002, rVS ρ = 0.33, *P* = .006), but not in session 1 in the reduced sample (lVS ρ = 0.08, *P* = .5, rVS ρ = 0.07, *P* = .55). Finally, changes in clinical scores did not correlate with changes in VS activity between sessions 1 and 2 (all *P*s > .36).

Correlations with DS and pPut activity can be found in supplementary analyses. Results showed that symptom variables, apart from the BACS, did not correlate with DS or pPut activity.

Substituting BNSS apathy and diminished expression for the PANSS negative factor did not significantly change the results: there was no association between VS activity and this factor (lVS *P* = .2; rVS *P* = .7).

Similarly, no correlations were observed between VS, DS, or pPut activity and the SNS apathy/diminished expression/total scores (all ρ between −0.051 and 0.1, all *P* > .12). The *P*-values reported are uncorrected for multiple comparisons.

#### Dimensional Relationships With Task Data.

In SZ at session 1, we found significant positive correlations between VS activity and response time speeding (ρ_SZ_SES1_lVS_RTSP_ = 0.51, *P* < .001; ρ_SZ_SES1_rVS_RTSP_ = 0.45, *P* < .001) and total reward amount (ρ_SZ_SES1_lVS_TRA_ = 0.50, *P* < .001; ρ_SZ_SES1_rVS_TRA_ = 0.41, *P* < .001). Similar positive correlations were found at session 2, between VS activity and response time speeding (ρ_SZ_SES2_lVS_RTSP_ = 0.36, *P* < .01; ρ_SZ_SES1_rVS_RTSP_ = 0.25, *P* < .05) and total reward amount (ρ_SZ_SES1_lVS_TRA_ = 0.36, *P* < .01; ρ_SZ_SES1_rVS_TRA_ = 0.26, *P* < .05). We found no correlation in SZ between VS activity and response time, nor between VS activity and accuracy at session 1 or session 2 (all *P*s > .12).

VS activity in HC at session 1 correlated positively with response time speeding (ρ_HC_SES1_lVS_RTSP_ = 0.35, *P* < .01; ρ_HC_SES1_rVS_RTSP_ = 0.32, *P* < .05) and negatively with accuracy (ρ_HC_SES1_lVS_ACC_ = −0.27, *P* < .05; ρ_HC_SES1_rVS_ACC_ = −0.35, *P* < .01). At session 2, VS activity in HC correlated positively with response time speeding, but solely in the rVS (ρ_HC_SES2_rVS_RTSP_ = 0.33, *P* < .05) but not in the lVS (ρ_HC_SES2_lVS_RTSP_ = 0.05, *P* = .74). We found no correlation between VS activity and response time or total reward amount at session 1 or session 2 (all *P*s > .17).

Correlations with DS and pPut activity were similar to correlations VS activity. Results can be found in supplementary analyses.

#### Controlling for Potential Confounds.

First, we controlled for behavioral metrics (response time speeding, accuracy, and the total reward amount) and cognition in our group difference analysis, comparing reward anticipation between groups and sessions and study sites. In the left VS, we observe a main effect of Group (*F*(131.3) = 5.2, *P* = .02, η_p_^2^ = 0.04, CI_95%_ = [0.0, 1]), as well as a main effect of speeding (*F*(228.9) = 6.5, *P* = .01, η_p_^2^ = 0.03, CI_95%_ = [0.0, 1]) and of total reward (*F*(220.8) = 3.98, *P* = .047, η_p_^2^ = 0.02, CI_95%_ = [0.0, 1]). There was also a Group by Session interaction (*F*(117.08) = 4.4, *P* = .04, η_p_^2^ = 0.03, CI_95%_ = [0.0, 1]), showing that patients improved between sessions (*P* = .046), whereas controls remained stable (*P* = .3), such that at session 2 there was no difference between the 2 groups (*P* = .3).

In the right VS we also observe the main effect of Group (*F*(135.8) = 11.07, *P* = .001, η_p_^2^ = 0.08, CI_95%_ = [0.02, 1]), as well as a main effect of speeding (*F*(230.6) = 6.8, *P* = .01, η_p_^2^ = 0.0096, CI_95%_ = [0.0, 1]). The main effect of total reward did not reach significance (*F*(205.3) = 3.8, *P* = .054, η_p_^2^ = 0.02, CI_95%_ = [0.0, 1]). No other effects approached significance.

Furthermore, partial Spearman correlations between reward anticipation in the lVS, rVS, and apathy were conducted, while including the most obvious potentially confounding variables: positive symptoms, medication, depression, cognition, and parkinsonism scores. They showed no change. Session 1: lVS ρ = 0.02, *P* = .87; rVS ρ = 0.13, *P* = .28. Session 2: lVS ρ = 0.09, *P* = .45; rVS ρ = 0.2, *P* = .12. Similar results to the above are observed for cognition (session 1: lVS ρ = 0.06, *P* = .7; rVS ρ = 0.09, *P* = .5. Session 2: lVS ρ = 0.39, *P* = .002; rVS ρ = 0.3, *P* = .02). No other significant correlations were found.

Finally, we controlled for the same covariates while performing ICC. We observe similar test-retest reliability, ie, moderate in controls (*ICC*_lVS_HC_ = 0.59 *ICC*_rVS_HC_ = 0.61) and poor in patients (*ICC*_lVS_SZ_ = 0.31 *ICC*_rVS_SZ_ = 0.39).

Thus, our main results hold after controlling for potential confounds.

## Discussion

The goal of this fMRI study was to evaluate ventral striatal hypoactivation during reward anticipation as a biomarker in patients with schizophrenia. As expected, we found decreased bilateral ventral striatal activity during reward anticipation in patients with schizophrenia compared to healthy controls. However, in contrast with our hypotheses, we found no association between ventral striatal activity in patients with schizophrenia and negative symptoms. Finally, we also showed that on the group level ventral striatal activity was stable over time and across centers in healthy controls and in patients with schizophrenia, ie, we found no changes between sessions or differences between centers in either patients or controls. However, individual retest reliability was poor in patients with schizophrenia.

### Hypoactivity During Reward Anticipation in Schizophrenia

In this study, we found decreased bilateral ventral striatal activation in patients with schizophrenia compared to healthy controls during reward anticipation. These results are in accordance with previous studies and meta-analyses, with similar or higher effect sizes.^4–6,15,42^

Additionally, we showed that ventral striatal activity in patients with schizophrenia and in healthy controls correlates with response time speeding, ie, both groups respond faster in higher reward trials. Such results have already been shown in healthy controls^43^ and in animal studies^44^ but to our knowledge not in patients with schizophrenia. The ventral striatum has been shown to play a significant role in reward anticipation by encoding the value of the future stimuli and increasing their salience to optimize goal-directed actions.^2,3^ Additionally, higher ventral striatal activity in patients with schizophrenia was also linked with higher monetary gains from the task. On the contrary, higher ventral striatal activity in healthy controls was associated with lower accuracy. This shows that patients with schizophrenia whose ventral striatum activates more tend to regulate their performance. However, when reward anticipation was intact, too much motivational salience could lead to increased willingness to respond, resulting in an inefficient response time/ accuracy trade-off.^45^

We also showed that these results may not be specific to the ventral striatum. The hypoactivation in SZ patients we found in the ventral striatum extends to the dorsal striatum and the posterior putamen. The dorsal striatum has been shown to be involved in reward anticipation deficits in schizophrenia in a previous meta-analysis.^6^ Here we showed that these regions, and especially the dorsal striatum, were also associated with response time speeding in both groups, which confirms their role in reward anticipation.^2,46^ In addition, the relationship between dorsal striatal activity and response time is consistent with the role of the dorsal striatum in action selection and its link with motor processes.^2,46^

Our exploratory whole-brain analyses added 2 other regions that show decreased activity during reward anticipation in patients with schizophrenia: the anterior cingulate cortex and the calcarine gyrus. Such decreased activation indicates that the salience and visual systems are also dysregulated during reward anticipation in schizophrenia.^47^

Taken together, our results confirm that the finding of ventral striatal hypoactivation in patients with schizophrenia during reward anticipation is robust. This reduction of activation extends beyond the ventral striatum to neighboring regions involved in motivational saliency and action selection.

### No Association Between Ventral Striatal Activity and Negative Symptoms in Schizophrenia

Contrary to our hypotheses, we found no association between ventral striatal activity in patients with schizophrenia and apathetic negative symptoms, or negative symptoms in general. This result goes against several meta-analyses and original studies including our own which found such a link.^4,6,10–12,42^ Several reasons should be taken into consideration to explain this divergence. First, the link between ventral striatal activity during reward anticipation and negative symptoms has shown a lot of variability including several negative findings in original studies.^14,15^ Meta-analyses confirm this variability and suggest that the association may be limited to the right ventral striatum.^4,6^ It has to be noted that some studies found a correlation between ventral striatal hypoactivation and positive symptoms.^14,15^

Finding an association between ventral striatal activity and negative symptoms might be modulated by several factors that may cause secondary negative symptoms, including the presence and severity of positive symptoms, medication dosage, and confounding symptoms like depression in the population assessed.^48^ All of these factors have been controlled in this study to assess primary negative symptoms as specifically as possible. Our patients did not score excessively high on negative symptoms. Thus, by excluding the most apathetic patients we potentially failed to capture the relationship between the symptoms and the brain response. Yet, the negative symptoms in the present study are in the range previously reported by studies finding the association.^4^ Other population characteristics may also play a role such as age and duration of illness. In any case, our results call into question the hypothesis that ventral striatal hypoactivity during reward anticipation is a robust neural marker of motivational negative symptoms. Future studies are needed to elucidate which factors determine the presence or absence of an association between negative symptoms and VS response to reward anticipation.

Future studies could explore if cortico-striatal functional dysconnectivity could be used as a more robust marker of negative symptoms, even in the absence of a link with ventral striatal activity (note, however, the limited test-retest reliability of functional connectivity measures^49^). Accordingly, we previously found that cortico-striatal functional dysconnectivity during reward anticipation correlated negatively with apathetic negative symptoms.^50^ Considering that a rapidly expanding number of studies has found global dysconnectivity patterns in schizophrenia^51–54^ and that cortico-striatal networks are known to modulate dopamine activity, they represent potential markers for early detection and treatment development.

### A Stronger Ventral Striatal Reward Anticipation Response Associated With Better Cognition in Schizophrenia

We find a correlation between the VS BOLD response during reward anticipation and patients’ cognitive scores: patients with better cognition showed a more intact reward brain response. Previous work has pointed toward an intimate link between cognition and reward processing in the general population, and their mutual impairment in schizophrenia, both in terms of behavior^55–57^ and brain circuits.^58–61^ In particular, impairments in the striatal function and striato-cortical interactions have been associated with both cognitive and motivational deficits.^62–64^ These impairments are thought to be bidirectional: motivational deficits may lead to cognitive impairments (eg, poor performance on cognitive tests due to reduced motivation^65,66^), and poor cognition may result in apathy (eg, through inaccurate reward value representation^67–69^). Interestingly, in our results, we find no correlations between striatal activity and apathy on the one hand, and no association between apathy and cognition, on the other. Thus, the mutual interactions postulated in the literature might show a certain asynchrony, may be limited only to some subfunctions, or be present in a subcategory of patients.

### Test-Retest Reliability and Stability Across Centers

We showed that ventral striatal activity during reward anticipation shows moderate test-rest reliability over time (ie, over the course of about 90 days) in healthy controls. Our values are within the range of MID reliability values in healthy controls from Grimm, Heinz^9^ and are well above values from previous meta-analysis on reliability in fMRI tasks^70,71^ suggesting that the MID task may have a relatively favorable profile in terms of retest reliability. For the first time, we investigated test-retest reliability of ventral striatal activity during reward anticipation in patients with schizophrenia. Retest reliability was poor, but the results are within the range of previous evaluations of reliability of fMRI tasks in schizophrenia^72^ and are close to values from the meta-analysis mentioned above.^70,71^ One possible explanation for the poor reliability found in patients is that there might be a difference in the way patients experienced sessions 1 and 2. Accordingly, session 2 might have been less stressful, since the patients already knew the environment, people, and tasks. Reproducing the fMRI tasks in a third session could have shown if that habituation effect did indeed take place.

In addition, we showed that ventral striatal activity in healthy controls and in patients with schizophrenia was stable across 2 different sites in Europe. This is the first indication that ventral striatal hypoactivity in schizophrenia can be reliably assessed across sites which is an important condition for its use as a biomarker in multicentric studies.

### Ventral Striatal Activity as a Neural Biomarker of Schizophrenia

Taken together, our results show that the MID robustly elicits group differences between patients with schizophrenia and healthy controls. Accordingly, we found new evidence that ventral striatal activity modulates motivational salience during reward anticipation to bias goal-directed actions toward rewarded stimuli. We also showed that the group difference between patients and controls was stable across centers.

However, 2 critical issues with respect to ventral striatal hypoactivation as a biomarker have been identified. While we showed that the difference between patients and controls was stable over 2 time points, individual scores were associated with only poor to moderate test-retest reliability. This is obviously a concern for clinical trials that want to include this measure for detection of target engagement during treatment or for prediction of treatment outcomes. To investigate this point, longitudinal studies with multiple assessments across longer time periods are needed to better characterize the evolution of ventral striatal hypoactivation over time. The second critical point concerns the absence of an association between ventral striatal activity in patients and apathy in our sample. While this may not be an issue if ventral striatal activation is considered as a biomarker of schizophrenia, it has to be kept in mind that negative symptoms are a domain of a largely unmet need where biomarkers may be most urgently needed. More research is therefore necessary to properly define the neural bases of apathy, and more generally, of negative symptoms.

A limitation to the present study is the use of a multiband sequence for data acquisition. While the present study was being conducted, research has shown that while offering several advantages, such sequences can induce signal loss in mesolimbic regions.^73^ Thus, the effect sizes we observe within the striatum may be reduced to some extent. We indeed observe reduced temporal signal-to-noise ratio in the striatum as compared to our previous work using single-band sequences (supplementary analysis 7.4). Importantly, however, the temporal signal-to-noise ratio did not show any correlation with the BOLD response within the striatum, thus suggesting a limited impact of the multiband sequence. Nevertheless, replication of the present results in a study with a single-band sequence would be of value.

## Conclusion

Our findings are consistent with the hypothesis that ventral striatal activity reflects modulation of motivational saliency during reward anticipation and that these processes are impaired in patients with schizophrenia. We provide additional evidence for the use of ventral striatal hypoactivity as a biomarker candidate for schizophrenia, but also raise critical issues that would need to be resolved before this measure can be considered as a biomarker, in particular with respect to negative symptoms.

## Supplementary Material

Supplementary material is available at https://academic.oup.com/schizophreniabulletin/.

sbae046_suppl_Supplementary_Materials

## References

[1] Knutson B , Westdorp A, Kaiser E, Hommer D. FMRI visualization of brain activity during a monetary incentive delay task. Neuroimage. 2000;12(1):20–27. 10875899 10.1006/nimg.2000.0593

[2] Oldham S , Murawski C, Fornito A, Youssef G, Yücel M, Lorenzetti V. The anticipation and outcome phases of reward and loss processing: a neuroimaging meta-analysis of the monetary incentive delay task. Hum Brain Mapp. 2018;39(8):3398–3418. 29696725 10.1002/hbm.24184 PMC6055646

[3] Wilson RP , Colizzi M, Bossong MG, Allen P, Kempton M, Bhattacharyya S; MTAC. The neural substrate of reward anticipation in health: a meta-analysis of fMRI findings in the monetary incentive delay task. Neuropsychol Rev. 2018;28(4):496–506. 30255220 10.1007/s11065-018-9385-5 PMC6327084

[4] Radua J , Schmidt A, Borgwardt S, et al. Ventral striatal activation during reward processing in psychosis: a neurofunctional meta-analysis. JAMA Psychiatry. 2015;72(12):1243–1251. 26558708 10.1001/jamapsychiatry.2015.2196

[5] Leroy A , Amad A, D'Hondt F, et al. Reward anticipation in schizophrenia: a coordinate-based meta-analysis. Schizophr Res. 2020;218:2–6. 31948895 10.1016/j.schres.2019.12.041

[6] Zeng J , Yan J, Cao H, et al. Neural substrates of reward anticipation and outcome in schizophrenia: a meta-analysis of fMRI findings in the monetary incentive delay task. Transl Psychiatry. 2022;12(1):448. 36244990 10.1038/s41398-022-02201-8 PMC9573872

[7] Vink M , de Leeuw M, Pouwels R, van den Munkhof HE, Kahn RS, Hillegers M. Diminishing striatal activation across adolescent development during reward anticipation in offspring of schizophrenia patients. Schizophr Res. 2016;170(1):73–79. 26631365 10.1016/j.schres.2015.11.018

[8] de Leeuw M , Kahn RS, Vink M. Fronto-striatal dysfunction during reward processing in unaffected siblings of schizophrenia patients. Schizophr Bull. 2014;41(1):94–103. 25368371 10.1093/schbul/sbu153 PMC4266310

[9] Grimm O , Heinz A, Walter H, et al. Striatal response to reward anticipation: evidence for a systems-level intermediate phenotype for schizophrenia. JAMA Psychiatry. 2014;71(5):531–539. 24622944 10.1001/jamapsychiatry.2014.9

[10] Kirschner M , Hager OM, Bischof M, et al. Ventral striatal hypoactivation is associated with apathy but not diminished expression in patients with schizophrenia. J Psychiatr Neurosci. 2016;41(3):152–161. 10.1503/jpn.140383 PMC4853206 26395814

[11] Stepien M , Manoliu A, Kubli R, et al. Investigating the association of ventral and dorsal striatal dysfunction during reward anticipation with negative symptoms in patients with schizophrenia and healthy individuals. PLoS One. 2018;13(6):e0198215. 29912880 10.1371/journal.pone.0198215 PMC6005482

[12] Wolf DH , Satterthwaite TD, Kantrowitz JJ, et al. Amotivation in schizophrenia: integrated assessment with behavioral, clinical, and imaging measures. Schizophr Bull. 2014;40(6):1328–1337. 24657876 10.1093/schbul/sbu026 PMC4193711

[13] Simon JJ , Biller A, Walther S, et al. Neural correlates of reward processing in schizophrenia—relationship to apathy and depression. Schizophr Res. 2010;118(1):154–161. 20005675 10.1016/j.schres.2009.11.007

[14] Esslinger C , Englisch S, Inta D, et al. Ventral striatal activation during attribution of stimulus saliency and reward anticipation is correlated in unmedicated first episode schizophrenia patients. Schizophr Res. 2012;140(1):114–121. 22784688 10.1016/j.schres.2012.06.025

[15] Nielsen MO , Rostrup E, Wulff S, et al. Alterations of the brain reward system in antipsychotic naïve schizophrenia patients. Biol Psychiatry. 2012;71(10):898–905. 22418013 10.1016/j.biopsych.2012.02.007

[16] Sheehan DV , Lecrubier Y, Sheehan KH, et al. The Mini-International Neuropsychiatric Interview (M.I.N.I.): the development and validation of a structured diagnostic psychiatric interview for DSM-IV and ICD-10. J Clin Psychiatry. 1998;59(suppl 20):22–33; quiz 33–34. 9881538

[17] Kirkpatrick B , Strauss GP, Nguyen L, et al. The brief negative symptom scale: psychometric properties. Schizophr Bull. 2011;37(2):300–305. 20558531 10.1093/schbul/sbq059 PMC3044634

[18] Strauss GP , Hong LE, Gold JM, et al. Factor structure of the brief negative symptom scale. Schizophr Res. 2012;142(1):96–98. 23062750 10.1016/j.schres.2012.09.007 PMC3502636

[19] Bischof M , Obermann C, Hartmann MN, et al. The brief negative symptom scale: validation of the German translation and convergent validity with self-rated anhedonia and observer-rated apathy. BMC Psychiatry. 2016;16(1):415. 27876020 10.1186/s12888-016-1118-9 PMC5118879

[20] Mucci A , Galderisi S, Merlotti E, et al; Italian Network for Research on Psychoses. The Brief Negative Symptom Scale (BNSS): independent validation in a large sample of Italian patients with schizophrenia. Eur Psychiatry. 2015;30(5):641–647. 25758156 10.1016/j.eurpsy.2015.01.014

[21] Kay SR , Fiszbein A, Opler LA. The positive and negative syndrome scale (PANSS) for schizophrenia. Schizophr Bull. 1987;13(2):261–276. 3616518 10.1093/schbul/13.2.261

[22] Wallwork RS , Fortgang R, Hashimoto R, Weinberger DR, Dickinson D. Searching for a consensus five-factor model of the Positive and Negative Syndrome Scale for schizophrenia. Schizophr Res. 2012;137(1):246–250. 22356801 10.1016/j.schres.2012.01.031 PMC3351536

[23] Addington D , Addington J, Maticka-tyndale E. Assessing depression in schizophrenia: the Calgary Depression Scale. Br J Psychiatry. 1993;163(S22):39–44. 8110442

[24] Gerlach J , Korsgaard S, Clemmesen P, et al. The St. Hans Rating Scale for extrapyramidal syndromes: reliability and validity. Acta Psychiatr Scand. 1993;87(4):244–252. 8098178 10.1111/j.1600-0447.1993.tb03366.x

[25] Keefe RSE , Goldberg TE, Harvey PD, Gold JM, Poe MP, Coughenour L. The Brief Assessment of Cognition in Schizophrenia: reliability, sensitivity, and comparison with a standard neurocognitive battery. Schizophr Res. 2004;68(2):283–297. 15099610 10.1016/j.schres.2003.09.011

[26] Leucht S , Samara M, Heres S, Davis JM. Dose equivalents for antipsychotic drugs: the DDD method. Schizophr Bull. 2016;42(suppl_1):S90–S94. 27460622 10.1093/schbul/sbv167 PMC4960429

[27] Simon JJ , Cordeiro SA, Weber M-A, et al. Reward system dysfunction as a neural substrate of symptom expression across the general population and patients with schizophrenia. Schizophr Bull. 2015;41(6):1370–1378. 26006262 10.1093/schbul/sbv067 PMC4601714

[28] Brainard DH. The psychophysics toolbox. Spatial Vis. 1997;10(4):433–436. 9176952

[29] Kleiner M , Brainard D, Pelli D. What’s new in Psychtoolbox-3? [ECVP 2007 abstract supplement]. Perception. 2007;36(14):1–16.

[30] Pelli DG. The VideoToolbox software for visual psychophysics: transforming numbers into movies. Spatial Vis. 1997;10:437–442. 9176953

[31] R Core Team. *R: A Language and Environment for Statistical Computing*. Vienna, Austria: R Foundation for Statistical Computing; 2019.

[32] Esteban O , Markiewicz CJ, Blair RW, et al. fMRIPrep: a robust preprocessing pipeline for functional MRI. Nat Methods. 2019;16(1):111–116. 30532080 10.1038/s41592-018-0235-4 PMC6319393

[33] Tustison NJ , Avants BB, Cook PA, et al. N4ITK: improved N3 bias correction. IEEE Trans Med Imaging. 2010;29(6):1310–1320. 20378467 10.1109/TMI.2010.2046908 PMC3071855

[34] Fonov VS , Evans AC, McKinstry RC, Almli CR, Collins DL. Unbiased nonlinear average age-appropriate brain templates from birth to adulthood. Neuroimage. 2009;47:S102.

[35] Avants BB , Epstein CL, Grossman M, Gee JC. Symmetric diffeomorphic image registration with cross-correlation: evaluating automated labeling of elderly and neurodegenerative brain. Med Image Anal. 2008;12(1):26–41. 17659998 10.1016/j.media.2007.06.004 PMC2276735

[36] Zhang Y , Brady M, Smith S. Segmentation of brain MR images through a hidden Markov random field model and the expectation-maximization algorithm. IEEE Trans Med Imaging. 2001;20(1):45–57. 11293691 10.1109/42.906424

[37] Cox RW. AFNI: software for analysis and visualization of functional magnetic resonance neuroimages. Comput Biomed Res. 1996;29(3):162–173. 8812068 10.1006/cbmr.1996.0014

[38] Jenkinson M , Bannister P, Brady M, Smith S. Improved optimization for the robust and accurate linear registration and motion correction of brain images. Neuroimage. 2002;17(2):825–841. 12377157 10.1016/s1053-8119(02)91132-8

[39] Greve DN , Fischl B. Accurate and robust brain image alignment using boundary-based registration. Neuroimage. 2009;48(1):63–72. 19573611 10.1016/j.neuroimage.2009.06.060 PMC2733527

[40] Abraham A , Pedregosa F, Eickenberg M, et al. Machine learning for neuroimaging with scikit-learn. Front Neuroinf. 2014:8–14. 10.3389/fninf.2014.00014 PMC3930868 24600388

[41] Mawlawi O , Martinez D, Slifstein M, et al. Imaging human mesolimbic dopamine transmission with positron emission tomography: I. Accuracy and precision of D2 receptor parameter measurements in ventral striatum. J Cereb Blood Flow Metab. 2001;21(9):1034–1057. 11524609 10.1097/00004647-200109000-00002

[42] Juckel G , Schlagenhauf F, Koslowski M, et al. Dysfunction of ventral striatal reward prediction in schizophrenia. Neuroimage. 2006;29(2):409–416. 16139525 10.1016/j.neuroimage.2005.07.051

[43] Insel C , Glenn CR, Nock MK, Somerville LH. Aberrant striatal tracking of reward magnitude in youth with current or past-year depression. J Abnorm Psychol. 2019;128(1): 44–56. 30489113 10.1037/abn0000389

[44] Roesch MR , Singh T, Brown PL, Mullins SE, Schoenbaum G. Ventral striatal neurons encode the value of the chosen action in rats deciding between differently delayed or sized rewards. J Neurosci. 2009;29(42):13365–13376. 19846724 10.1523/JNEUROSCI.2572-09.2009 PMC2788608

[45] Leong JK , MacNiven KH, Samanez-Larkin GR, Knutson B. Distinct neural circuits support incentivized inhibition. Neuroimage. 2018;178:435–444. 29803959 10.1016/j.neuroimage.2018.05.055 PMC6398995

[46] Balleine BW , Delgado MR, Hikosaka O. The role of the dorsal striatum in reward and decision-making. J Neurosci. 2007;27(31):8161–8165. 17670959 10.1523/JNEUROSCI.1554-07.2007 PMC6673072

[47] Schneider M , Leuchs L, Czisch M, Sämann PG, Spoormaker VI. Disentangling reward anticipation with simultaneous pupillometry/ fMRI. Neuroimage. 2018;178:11–22. 29733957 10.1016/j.neuroimage.2018.04.078

[48] Hägele C , Schlagenhauf F, Rapp M, et al. Dimensional psychiatry: reward dysfunction and depressive mood across psychiatric disorders. Psychopharmacology (Berl). 2015;232(2):331–341. 24973896 10.1007/s00213-014-3662-7 PMC4297301

[49] Noble S , Scheinost D, Constable RT. A decade of test-retest reliability of functional connectivity: a systematic review and meta-analysis. Neuroimage. 2019;203:116157. 31494250 10.1016/j.neuroimage.2019.116157 PMC6907736

[50] Carruzzo F , Kaiser S, Tobler PN, Kirschner M, Simon JJ. Increased ventral striatal functional connectivity in patients with schizophrenia during reward anticipation. Neuroimage Clin. 2022;33:102944. 35078045 10.1016/j.nicl.2022.102944 PMC8789684

[51] Li T , Wang Q, Zhang J, et al. Brain-wide analysis of functional connectivity in first-episode and chronic stages of schizophrenia. Schizophr Bull. 2016;43(2):sbw099–sbw448. 10.1093/schbul/sbw099 PMC5605268 27445261

[52] Pettersson-Yeo W , Allen P, Benetti S, McGuire P, Mechelli A. Dysconnectivity in schizophrenia: where are we now? Neurosci Biobehav Rev. 2011;35(5):1110–1124. 21115039 10.1016/j.neubiorev.2010.11.004

[53] Fornito A , Harrison BJ, Goodby E, et al. Functional dysconnectivity of corticostriatal circuitry as a risk phenotype for psychosis. JAMA Psychiatry. 2013;70(11):1143–1151. 24005188 10.1001/jamapsychiatry.2013.1976

[54] Sabaroedin K , Tiego J, Fornito A. Circuit-based approaches to understanding corticostriatothalamic dysfunction across the psychosis continuum. Biol Psychiatry. 2023;93(2):113–124. 36253195 10.1016/j.biopsych.2022.07.017

[55] Beck AT , Rector NA. Cognitive approaches to schizophrenia: theory and therapy. Annu Rev Clin Psychol. 2005;1:577–606. 17716100 10.1146/annurev.clinpsy.1.102803.144205

[56] Grant PM , Beck AT. Defeatist beliefs as a mediator of cognitive impairment, negative symptoms, and functioning in schizophrenia. Schizophr Bull. 2009;35(4):798–806. 18308717 10.1093/schbul/sbn008 PMC2696369

[57] Reddy LF , Horan WP, Barch DM, et al. Understanding the association between negative symptoms and performance on effort-based decision-making tasks: the importance of defeatist performance beliefs. Schizophr Bull. 2018;44(6):1217–1226. 29140501 10.1093/schbul/sbx156 PMC6192468

[58] Kahn I , Shohamy D. Intrinsic connectivity between the hippocampus, nucleus accumbens, and ventral tegmental area in humans. Hippocampus. 2013;23(3):187–192. 23129267 10.1002/hipo.22077 PMC4118056

[59] Diekhof EK , Gruber O. When desire collides with reason: functional interactions between anteroventral prefrontal cortex and nucleus accumbens underlie the human ability to resist impulsive desires. J Neurosci. 2010;30(4):1488–1493. 20107076 10.1523/JNEUROSCI.4690-09.2010 PMC6633806

[60] Huskey R , Craighead B, Miller MB, Weber R. Does intrinsic reward motivate cognitive control? A naturalistic-fMRI study based on the synchronization theory of flow. Cogn Affect Behav Neurosci. 2018;18:902–924. 29923098 10.3758/s13415-018-0612-6

[61] Horne CM , Vanes LD, Verneuil T, et al. Cognitive control network connectivity differentially disrupted in treatment resistant schizophrenia. Neuroimage Clin. 2021;30:102631. 33799270 10.1016/j.nicl.2021.102631 PMC8044714

[62] Harvey PD , Koren D, Reichenberg A, Bowie CR. Negative symptoms and cognitive deficits: what is the nature of their relationship? Schizophr Bull. 2006;32(2):250–258. 16221995 10.1093/schbul/sbj011 PMC2632205

[63] Robison A , Thakkar KN, Diwadkar VA. Cognition and reward circuits in schizophrenia: synergistic, not separate. Biol Psychiatry. 2020;87(3):204–214. 31733788 10.1016/j.biopsych.2019.09.021 PMC6946864

[64] Simpson EH , Kellendonk C, Kandel E. A possible role for the striatum in the pathogenesis of the cognitive symptoms of schizophrenia. Neuron. 2010;65(5):585–596. 20223196 10.1016/j.neuron.2010.02.014 PMC4929859

[65] Fervaha G , Zakzanis KK, Foussias G, Graff-Guerrero A, Agid O, Remington G. Motivational deficits and cognitive test performance in schizophrenia. JAMA Psychiatry. 2014;71(9):1058–1065. 25075930 10.1001/jamapsychiatry.2014.1105

[66] Fervaha G , Agid O, Foussias G, Remington G. Effect of intrinsic motivation on cognitive performance in schizophrenia: a pilot study. *Schizophrenia Res*. 2014;152:317–318. 10.1016/j.schres.2013.11.037 24333003

[67] Heerey EA , Bell-Warren KR, Gold JM. Decision-making impairments in the context of intact reward sensitivity in schizophrenia. Biol Psychiatry. 2008;64(1):62–69. 18377874 10.1016/j.biopsych.2008.02.015 PMC2613513

[68] Heerey EA , Robinson BM, McMahon RP, Gold JM. Delay discounting in schizophrenia. Cogn Neuropsychiatry. 2007;12(3):213–221. 17453902 10.1080/13546800601005900 PMC3746343

[69] Weickert TW , Goldberg TE, Callicott JH, et al. Neural correlates of probabilistic category learning in patients with schizophrenia. J Neurosci. 2009;29(4):1244–1254. 19176832 10.1523/JNEUROSCI.4341-08.2009 PMC2775494

[70] Elliott ML , Knodt AR, Ireland D, et al. What is the test-retest reliability of common task-functional MRI measures? New empirical evidence and a meta-analysis. Psychol Sci. 2020;31(7):792–806. 32489141 10.1177/0956797620916786 PMC7370246

[71] Bennett CM , Miller MB. How reliable are the results from functional magnetic resonance imaging? Ann N Y Acad Sci. 2010;1191(1):133–155. 20392279 10.1111/j.1749-6632.2010.05446.x

[72] Manoach DS , Halpern EF, Kramer TS, et al. Test-retest reliability of a functional MRI working memory paradigm in normal and schizophrenic subjects. Am J Psychiatry. 2001;158(6):955–958. 11384907 10.1176/appi.ajp.158.6.955

[73] Srirangarajan T , Mortazavi L, Bortolini T, Moll J, Knutson B. Multi-band FMRI compromises detection of mesolimbic reward responses. Neuroimage. 2021;244:118617. 34600102 10.1016/j.neuroimage.2021.118617 PMC8626533

