# Supplementary material for: Striatal Response to Reward Anticipation as a Biomarker for Schizophrenia and Negative Symptoms: Effects, Test-Retest Reliability, and Stability Across Sites
Source: Schizophr Bull. 2024 Apr 19;50(4):733–46. doi: 10.1093/schbul/sbae046 (PMC11283203; doi:10.1093/schbul/sbae046)

6. Supplementary Methods

6.1 The SYMONE Cohort

The analyses presented here were performed on the SYMONE cohort. The SYMONE cohort is a clinical cohort including patients with schizophrenia and healthy controls. The SYMONE cohort was designed to define behavioral and neural markers of apathy in schizophrenia. The definition of such markers depends on three main points: 1) a robust association between markers and apathy that has 2) longitudinal stability and 3) stability across centers. The design of the study was also done to test the predictive power of these biomarkers on apathy levels after nine months. The SYMONE study took place over three sessions (Session 1, Session 2 after three months and Session 3 after six months) in two centers (Berlin, Germany and Geneva, Switzerland). The study comprised a full demographical and clinical evaluation, two behavioral tasks and two functional magnetic resonance imaging (fMRI) tasks (Supplementary Table 1). Sessions 1 and 2 were composed of all three parts, while the Session 3 comprised solely the demographical and clinical evaluation. The study was approved by the local ethics committees in both centers.

Patients with schizophrenia were recruited from outpatient units at the University Hospital in Geneva, Switzerland and at the Charité Hospital in Berlin, Germany. Inclusion criteria included a diagnosis of schizophrenia, a clinical stability, and no hospitalization or medication change in the last four months. Major causes for secondary negative symptoms were excluded (i.e. current major depressive episode, florid psychotic symptoms and extra-pyramidal side-effects). Healthy control participants were recruited from the general population and were matched with the patient group based on their age, gender and personal and parental education. Healthy controls with a history of psychiatric or neurologic disorders were excluded. Power analyses indicated that a sample of 66 patients with schizophrenia and 32 healthy controls (with an estimated drop-out rate of 30% due to the longitudinal design of the study) per center was necessary. Due to the Covid19 pandemic, we failed to recruit that many participants. At the end of the recruitment phase, we had included 60 patients and 32 healthy controls in Geneva and 47 patients and 36 healthy controls in Berlin. Note that participant numbers in articles based on the SYMONE cohort may vary, as not all participants performed every task in every session (see Supplementary Figure 1 for the participants inclusion in this study).

7. Supplementary Analyses

7.1 Supplementary ROI Analyses

Additional mixed models identified a significant effect of Group, with HC having more activity than SZ in lDS (*M_lDS_HC_* = 1.28, *SE_lDS_HC_* = .11; *M_lDS_SZ_* = .37, *SE_lDS_SZ_* = .10; *F*(122) = 40.06, *p* < .001), lpPut (*M_lpPut_HC_* = 1.08, *SE_lpPut_HC_* = .09; *M_lpPut_SZ_* = .25, *SE_lpPut_SZ_* = .08; *F*(122) = 46.76, *p* < .001), rDS (*M_rDS_HC_* = 1.38, *SE_rDS_HC_* = .12; *M_rDS_SZ_* = .43, *SE_rDS_SZ_* = .11; *F*(122) = 37.94, *p* < .001), rpPut (*M_rpPut_HC_* = .89, *SE_rpPut_HC_* = .09; *M_rpPut_SZ_* = .12, *SE_rpPut_SZ_* = .08; *F*(122) = 43.66, *p* < .001).There was also an interaction effect between Group and Session in rDS activity, where SZ had lower activity in Session 1 (*M_lDS_SZ_SES1_* = .22, *SE_lDS_SZ_SES1_* = .11) than in Session 2 (*M_lDS_SZ_SES2_* = .57, *SE_lDS_SZ_SES2_* = .21; *t*(122) = -2.53, *p* < .05). No other effect was detected (all *ps* > .06).

Dorsal striatal activity was stable across Session 1 and Session 2 for SZ (*ICC_lDS_SZ_* = .25, *p* < .05; *ICC_rDS_SZ_* = .31, *p* < .05) and HC (*ICC_rDS_HC_* = .46, *p* < .05; *ICC_lDS_HC_* = .48, *p* < .05). While posterior putamen was stable for HC (*ICC_rpPut_HC_* = .35, *p* < .05; *ICC_lpPut_HC_* = .30, *p* < .05), but not for SZ (*ICC_lpPut_SZ_* = .06, *p* = .31; *ICC_rpPut_SZ_* = .17, *p* = .08).

7.2 Supplementary Dimensional Analyses

Complementary analyses in SZ at Session 1 showed similar positive correlations between DS activity and response time speeding (*ρ_SZ_SES1_lDS_RTSP_* = .38, *p* < .01; *ρ_SZ_SES1_rDS_RTSP_* = .38, *p* < .01) and total reward amount (*ρ_SZ_SES1_lDS_TRA_* = .35, *p* < .01; *ρ_SZ_SES1_rDS_TRA_* = .38, *p* < .01), though the relation is less clear with pPut activity as only the left ROI correlated positively with response time speeding (*ρ_SZ_SES1_lpPut_RTSP_* = .26, *p* < .05; *ρ_SZ_SES1_rpPut_RTSP_* = .14, *p* = .26; *ρ_SZ_SES1_lpPut_TRA_* = .25, *p* < .05; *ρ_SZ_SES1_rpPut_TRA_* = .17, *p* = .18). At Session 2, DS activity correlated positively with total reward amount (*ρ_SZ_SES2_lDS_TRA_* = .38, *p* < .01; *ρ_SZ_SES2_rDS_TRA_* = .38, *p* < .01) and lDS activity correlated negatively with response time (*ρ_SZ_SES2_lDS_RT_* = -.25, *p* < .05). lpPut activity correlated positively with total reward amount (*ρ_SZ_SES2_lpPut_TRA_* = .25, *p* < .05). No other association was significant (all *ps* > .10).

In HC, complementary analyses at Session 1 showed positive correlations between DS activity and response time speeding (*ρ_HC_SES1_lDS_RTSP_* = .28, *p* < .05; *ρ_HC_SES1_rDS_RTSP_* = .32, *p* < .05). lpPut activity also correlated positively with response time speeding (*ρ_HC_SES1_lpPut_RTSP_* = .32, *p* < .05). All other correlations were not significant (all *ps* > .08). At Session 2, DS activity in HC correlated only with response time speeding (*ρ_HC_SES2_lDS_RTSP_* = .32, *p* < .05; *ρ_HC_SES2_rDS_RTSP_* = .30, *p* < .05). A similar relationship was found with pPut and response time speeding (*ρ_HC_SES2_lpPut_RTSP_* = .28, *p* < .05; *ρ_HC_SES2_rpPut_RTSP_* = .35, *p* < .01). No other correlation was significant (all *ps* > .10).

7.3 Combining High Reward and Low Reward

Our approach in the main text followed our previous articles by reporting only the High reward > No reward contrast (i.e., the contrast which produced the strongest reward-related anticipation response). In an exploratory approach, we ran a set of analyses on the contrast Reward > No Reward, where “Reward” combined trials in the high and low reward conditions.

Similar to the result reported in the manuscript, mixed effect model on both the left and the right VS only showed a main effect of Group, with HC showing a stronger response, compared to SZ. Left VS: *M_lVS_HC_* = 1.18, *SE_lVS_HC_* = .27 (session 1), *M_lVS_HC_* = 1.19, *SE_lVS_HC_* = .24 (session 2); *M_lVS_SZ_* = .005, *SE_lVS_SZ_* = .2 (session 1), *M_lVS_SZ_* = .36, *SE_lVS_SZ_* = .21; *F*(118) = 13.14, *p* = .0004. No other effects reached significance: all *F*<2.35, all *p*> .24. Right VS: *M_lVS_HC_* = 1.4, *SE_lVS_HC_* = .28 (session 1), *M_lVS_HC_* = 1.49, *SE_lVS_HC_* = .23 (session 2); *M_lVS_SZ_* = .19, *SE_lVS_SZ_* = .22 (session 1), *M_lVS_SZ_* = .47, *SE_lVS_SZ_* = .22 *F*(118) = 13.95, *p* = .0003. No other effects reached significance: all *F*<3.34, all *p*> .07. Thus, the result was very similar to that including only high reward trials that is reported in the manuscript (left – lVS, right – rVS).

We calculated intra-class correlation coefficients to assess test-retest reliability, which appeared similar to those reported in the manuscript for the high vs no reward contrast (i.e. poor for patients and moderate for controls):

|  | **New ICC** | | **ICC in main text** | |
| --- | --- | --- | --- | --- |
|  | **Left VS** | **Right VS** | **Left VS** | **Right VS** |
| **Controls** | 0.53 | 0.57 | 0.56 | 0.65 |
| **Patients** | 0.38 | 0.48 | 0.36 | 0.48 |

Finally, we find no correlations between reward anticipation in the ventral striatum and apathy in either Session 1 (*ρ_lVS_* = .0025, *p* = .98; *ρ_rVS_* = .095, *p* = .45) or Session 2 (*ρ_lVS_* = .057, *p* = .65; *ρ_rVS_* = .068, *p* = .58).

7.4 Temporal Signal-to-Noise Ratio Analyses

To evaluate the extent to which the multi-band sequence we used resulted in signal loss in the striatum, we compared our participants’ temporal signal-to-noise ratio (tSNR) with that of participants from previous work which used single-band sequences and the MID task. We calculated the tSNR by taking the mean of the time series within the ROIs and dividing them by the standard deviations of these mean time series. The functional data was resliced and normalised (but not smoothed). The tSNR thus represents the average signal level relative to the variability of the mean signal. We compared four ROIs (left VS, left DS and left fusiform face area) between the following datasets (only healthy controls were included): SYMONE (N=55), Kirschner et al., 2016 (N=25) and Carruzzo et al., 2023 (N=84).

We show reduced signal in SYMONE participants in both the lVS and lDS as compared to the other two datasets (ROI*dataset interaction *F*(4,789.51)=26.9, *p* < .0001; Left ventral striatum: SYMONE-Kirschner: *p* < .0001, SYMONE-Carruzzo: *p* = .0007; Left dorsal striatum: SYMONE-Kirschner: *p* < .0001, SYMONE- Carruzzo: *p* = .0091), but no differences between datasets on tSNR in the FFA (SYMONE-Kirschner: *p* = .96; SYMONE- Carruzzo: *p* = .08). This result confirms reduced SNR in the striatum when activity is measures with a multiband sequence, as compared to preserved tSNR in other regions (Supplementary Figure 4).

We also assessed whether there was a relationship between this loss of signal and the reward anticipation response. We find no correlations between tSNR and the reward anticipation response in either Session 1 the left (*ρ* = .14, *p* = .14) or the right (*ρ* = .16, *p* = .083) VS, not in Session 2 the left (*ρ* = .14, *p* = .14) or the right (*ρ* = .16, *p* = .083) VS.

Finally, we evaluated whether this loss of signal affected the stability of the reward anticipation response over time. We averaged the tSNR across the two sessions for each participant and correlated it with the absolute value of ventral striatum mean reward anticipation signal change between Session 1 and 2. We find no correlation**s** for either the right (*ρ* = .057, *p* = .53) or the left VS (*ρ* = .1, *p* = .25).

7.5 Assessing the Potential Influence of Motion

To assess any residual effects of motion on our results we calculated framewise displacement (FD) for each participant prior to removing any volumes and performed Spearman correlations between these values and our main contrast [high reward – no reward]. We find no correlations for either patients or controls, in either the left (HC: *ρ =* -.098, *p* = .48; SZ: *ρ =* .13, *p* = .3) or the right (HC: *ρ =* -.045, *p* = .74; SZ: *ρ =* .14, *p* = .24) VS. In the full sample, FD across both sessions did not correlate with the absolute value of the difference between the reward anticipation signal at Session 1 and 2, i.e. we find no correlation of average FD with activity change in the left (*ρ =* .11, *p* = .23) or the right (*ρ =* .004, *p* = .96) VS.

We also included FD in partial correlations between right and left VS activity and BNSS apathy and diminished expression. We find no significant correlations (rVS and BNSS Apathy: *ρ* = .15, p = .43; between lVS and BNSS Apathy: *ρ* = -.11, p = .56).

Finally, we introduced FD as a covariate for our group differences analyses. In both the left (*F*(128.7) = 24.8, *p* < .0001) and the right (*F*(127.2) = 16.5, *p* < .0001) VS we find a main effect of Group as reported in the main text (main effect of FD: in the lVS *F*(195.4) = 0.09, *p* = .76, in the rVS *F*(212.4) = 1.85, *p* = .2. Similar to the main text results, rhere were no other main effects nor interactions (all *F* < 3.4, all *p* > .067).

7.6 Assessing the Spatial Activation Overlap between Sites

To test for any spatial site differences, we calculated the Dice overlap index in the VS ROI used at different statistical thresholds (1) *Z* = 1.64 (*p* < 0.05 uncorrected), 2) *Z* = 2.3 (*p* < 0.01 uncorrected) 3) *Z* = 3.09 (*p* < 0.001 uncorrected), and 4) *Z* > 4.0, (~ *p* < 10^− 4^, which is quite conservative). We show very strong overlap in both VS regions:

| **Right VS** | Z=1.64 | Z=2.3 | Z=3.09 | Z=4 |
| --- | --- | --- | --- | --- |
| Nb overlapping voxels | 134 | 123 | 99 | 59 |
| Nb active voxels Geneva | 134 | 123 | 100 | 60 |
| Nb active voxels Berlin | 139 | 136 | 122 | 100 |
| Overlap ratio | 0.98 | 0.95 | 0.89 | 0.74 |

| **Left VS** | Z=1.64 | Z=2.3 | Z=3.09 | Z=4 |
| --- | --- | --- | --- | --- |
| Nb overlapping voxels | 158 | 133 | 92 | 49 |
| Nb active voxels Geneva | 163 | 141 | 99 | 56 |
| Nb active voxels Berlin | 182 | 170 | 150 | 112 |
| Overlap ratio | 0.92 | 0.86 | 0.74 | 0.58 |

We are thus quite confident in the absence of Site effects for reward anticipation.

7.7 Using a Different Ventral Striatum ROI

To target the ventral striatum more specifically we used a smaller ROI of the nucleus accumbens derived from the WFU PickAtlas, and previously used in Katthagen et al., 2018 (Supplementary figure 4). We performed similar analyses to the main text and observe the same results. In terms of differences in brain activity across groups and sessions, there was a main effect of Group in both the left (*M_lVS_HC_* = 1.37, *SE_lVS_HC_* = .16; *M_lVS_SZ_* = .62, *SE_lVS_SZ_* = .12; *F*(122)=11.1, *p* = .001) and the right (*M_rVS_HC_* = 1.3, *SE_rVS_HC_* = .13; *M_rVS_SZ_* = .4, *SE_rVS_SZ_* = .1; *F*(122)=20.12, *p* < .0001) NAcc. No other effect reached significance (lVS all *F*<1.55, all *p* > .2; rVS all *F*< 3.03, all *p* > .08).

There were no correlations between VS activity and BNSS apathy at Session 1 (lVS *ρ* = -.01, *p* = .93; rVS *ρ* = .23, *p*=.06 – this trend level correlation was due to an outlier, after removing the outlier it became rVS *ρ* = .2, *p* =.1), or at Session 2 (lVS *ρ* = .04, *p* = .75; rVS *ρ* = .05, *p* =.7). We again observe a correlation with cognition, as measured by the BACS (lVS *ρ* = .41, *p* < .001; rVS *ρ* = .24, *p* =.047).

Supplementary Tables

| **Supplementary Table 1.** Summary of the Data Acquired for Participants in the SYMONE Cohort | | |
| --- | --- | --- |
| **Demographics** (including age, gender, education of participant and parents, onset and duration of illness, medication) | | |
| **Clinical Evaluation** | |  |
|  | Major psychiatric disorders evaluation | Mini-International Neuropsychiatric Interview (MINI; Sheehan et al., 1998) |
|  | Negative symptoms (hetero-evaluation) | Brief Negative Symptom Scale (BNSS; Kirkpatrick et al., 2011; Strauss et al., 2012) |
|  | Positive and negative symptoms (hetero-evaluation) | Positive and Negative Syndrome Scale (PANSS; Kay, Fiszbein, & Opler, 1987) |
|  | Negative symptoms (self-evaluation) | Self-evaluation of Negative Symptoms (SNS; Dollfus et al., 2016) |
|  | Apathy | Marins Apathy Scale for clinicians (AES; Marins, 1991) |
|  | Motivation | Demotivational Beliefs Inventory (DBI; Pillny et al., 2018) |
|  | Depressive symptoms | Calgary Depression Scale (CDS; Addington, Addington, & Maticka-tyndale, 1993) |
|  | Cognition score | Brief Assessment of Cognition in Schizophrenia (BACS; Keefe, 2004) |
|  | Reaction to ambiguous situations | Intolerance of Uncertainty Scale (IUS; Freestone, 1994) |
|  | Parkinsonism symptoms | St. Hans Rating Scale (SHRS; Gerlach et al., 1993) |
|  | Global functioning | Personal and Social Performance scale (PSP; Morosini, 2000) |
|  |  | Global Assessment of Functioning scale (GAF; Frances, 1994) |
| **Behavioral Tasks** | |  |
|  | Physical effort-based decision making task | Effort-based Decision Making task (Hartmann et al., 2015) |
|  | Reinforcement learning | One-Step task (similar to Frank et al., 2003) |
| **fMRI Tasks** | |  |
|  | Reward anticipation | Monetary Incentive Delay task (MID; Knutson, 2000) |
|  | Reinforcement learning | Volatile Reversal task (similar to Boehme et al., 2015) |
|  |  |  |

| **Supplementary Table 2.** Clinical Characteristics of Patients with Schizophrenia and Healthy Controls at Session 2 | | | | | | | | | | | | | | | | |
| --- | --- | --- | --- | --- | --- | --- | --- | --- | --- | --- | --- | --- | --- | --- | --- | --- |
|  |  | Session 2 | | | | | | | | | | | | | | |
|  |  | Berlin | | |  | Geneva | | |  | All | | |  | Statistics | | |
|  |  | SZ (N=28) |  | HC (N=26) |  | SZ (N=39) |  | HC (N=29) |  | SZ (N=67) |  | HC (N=55) |  |  |  |  |
|  |  |  |  |  |  |  |  |  |  |  |  |  |  | CH vs GE | | |
|  |  |  |  |  |  |  |  |  |  |  |  |  |  |  |  |  |
| **Clinical Variables** | |  |  |  |  |  |  |  |  |  |  |  |  | SZ |  | HC |
| BNSS | |  |  |  |  |  |  |  |  |  |  |  |  |  |  |  |
|  | Apathy | 16.9 (1.93) |  | 1.77 (0.49) |  | 12.0 (1.29) |  | 0.517 (0.19) |  | 14.1 (1.13) |  | 1.11 (0.26) |  | *W = 713*, * |  |  |
|  | Diminished Expression | 9.11 (1.29) |  | 0.808 (0.31) |  | 7.21 (1.02) |  | 0.828 (0.32) |  | 8.00 (0.81) |  | 0.818 (0.23) |  |  |  |  |
|  | Total | 26.0 (2.95) |  | 2.58 (0.58) |  | 19.2 (2.05) |  | 1.34 (0.36) |  | 22.1 (1.75) |  | 1.93 (0.34) |  | *W = 704.5*, * |  |  |
| PANSS | |  |  |  |  |  |  |  |  |  |  |  |  |  |  |  |
|  | Negative Factor | 16.4 (1.34) |  | 7.00 (0.31) |  | 13.4 (0.91) |  | 6.31 (0.14) |  | 14.6 (0.79) |  | 6.64 (0.17) |  |  |  |  |
|  | Positive Factor | 7.29 (0.68) |  | 4.15 (0.09) |  | 5.77 (0.39) |  | 4.14 (0.08) |  | 6.40 (0.38) |  | 4.15 (0.06) |  |  |  |  |
|  | Total | 57.3 (3.36) |  | 32.7 (0.62) |  | 45.6 (1.54) |  | 30.7 (0.23) |  | 50.5 (1.80) |  | 31.7 (0.34) |  | *W = 749.5*, ** |  | *W = 506*, * |
| CDS Total | | 3.21 (0.70) |  | 0.85 (0.25) |  | 2.08 (0.42) |  | 0.24 (0.13) |  | 2.55 (0.39) |  | 0.53 (0.14) |  |  |  | *W = 486*, * |
| BACS Total (z score) | | -0.94 (0.26) |  | 0.66 (0.25) |  | -1.59 (0.18) |  | 0.89 (0.23) |  | -1.32 (0.16) |  | 0.78 (0.17) |  | *W = 711*, * |  |  |
| SHRS Parkinsonism Score | | 2.07 (0.83) |  |  |  | 3.95 (0.78) |  |  |  | 3.16 (0.58) |  |  |  | *W = 306.5*, ** |  |  |
| RIS Equivalence | | 5.23 (0.73) |  |  |  | 4.83 (0.42) |  |  |  | 5.00 (0.39) |  |  |  |  |  |  |
|  |  |  |  |  |  |  |  |  |  |  |  |  |  |  |  |  |
| Scores displayed as Mean (SE). *** *p* < .001, ** *p* < .01, * *p* < .05 | | | | | | | | | | | | |  |  |  |  |

| **Supplementary Table 3.** Stability of Clinical Characteristics of Patients with Schizophrenia and Healthy Controls between Session 1 and Session 2 | | | | |
| --- | --- | --- | --- | --- |
|  |  | Session 1 & Session 2 | | |
|  |  | ICC(A,1) | | |
| **Clinical Variables** | | SZ |  | HC |
| BNSS | |  |  |  |
|  | Apathy | .88 |  | .76 |
|  | Diminished Expression | .88 |  | .83 |
|  | Total | .90 |  | .80 |
| PANSS | |  |  |  |
|  | Negative Factor | .84 |  | .75 |
|  | Positive Factor | .81 |  | .71 |
|  | Total | .90 |  | .65 |
| CDS Total | | .72 |  | .33 |
| BACS Total (z score) | | .83 |  | .31 |
| SHRS Parkinsonism Score | | .81 |  |  |
| RIS Equivalence | | .85 |  |  |
|  |  |  |  |  |
|  |  |  |  |  |

| **Supplementary Table 4.** Whole-Brain Localized Analysis Results for the Contrast High Reward > No Reward Anticipation in Session 1 and Session 2 | | | | | | | | |
| --- | --- | --- | --- | --- | --- | --- | --- | --- |
| Session | Side | Structures | MNI Coordinates | | | | t | Number of Voxels |
|  |  |  | x | y | z |  |  |  |
| Session 1 | Left | Posterior Medial Thalamus | -4 | -26 | 0 |  | 7.32 | 67 |
|  |  | Putamen | -20 | 10 | -6 |  | 6.83 | 573 |
|  |  | Ventral Striatum |  |  |  |  |  |  |
|  |  | Dorsal Striatum |  |  |  |  |  |  |
|  |  | Amygdala |  |  |  |  |  |  |
|  |  | Thalamus (Right) |  |  |  |  |  |  |
|  |  | Thalamus | -14 | -16 | -4 |  | 6.55 | 51 |
|  |  | Thalamus | -8 | -20 | 12 |  | 6.46 | 30 |
|  |  | Anterior Cingulate Cortex | -6 | 14 | 34 |  | 6.22 | 37 |
|  |  | Calcarine Gyrus | -10 | -84 | 6 |  | 6.02 | 73 |
|  | Right | Putamen | 18 | 6 | 10 |  | 7.25 | 645 |
|  |  | Ventral Striatum |  |  |  |  |  |  |
|  |  | Dorsal Striatum |  |  |  |  |  |  |
|  |  | Amygdala |  |  |  |  |  |  |
| Session 2 | Left | Putamen | -20 | 8 | -6 |  | 5.94 | 475* |
|  |  | Ventral Striatum |  |  |  |  |  |  |
|  |  | Dorsal Striatum |  |  |  |  |  |  |
|  |  | Amygdala |  |  |  |  |  |  |
|  |  | Thalamus (Right) |  |  |  |  |  |  |
|  | Right | Putamen | 24 | 2 | -10 |  | 5.03 | 495* |
|  |  | Ventral Striatum |  |  |  |  |  |  |
|  |  | Dorsal Striatum |  |  |  |  |  |  |
|  |  | Amygdala |  |  |  |  |  |  |
| Note. Unmarked results are p<.05 FWE corrected at the cluster level for the whole brain (underlying height threshold: p<.05, FWE corrected, threshold at 30 voxels). *p<.05 FWE corrected at the cluster level for the masked brain (underlying height threshold: p<.001 uncorrected, threshold at 30 voxels). | | | | | | | | |

| **Supplementary Table 5**. Mean left and right ventral striatum activation by experimental Site  in each Session and Group | | | | | |
| --- | --- | --- | --- | --- | --- |
| Session | Site |  | Group | mean | SD |
| 1 | GE |  | SZ | 0.67023608 | 1.244964 |
| 1 | GE |  | HC | 1.55154075 | 1.768448 |
| 1 | CH |  | SZ | 0.09532076 | 1.098590 |
| 1 | CH |  | HC | 1.56534751 | 1.504331 |
| 2 | GE |  | SZ | 0.88028020 | 1.277237 |
| 2 | GE |  | HC | 1.28157364 | 1.700006 |
| 2 | CH |  | SZ | 0.56573969 | 1.245477 |
| 2 | CH |  | HC | 1.54984312 | 1.295403 |

GE = Germany, CH = Switzerland; SZ – patients with schizophrenia, HC – healthy controls

| Medication | Nb patients |
| --- | --- |
| Amisulprid | 13 |
| Aripiprazol | 21 |
| Clozapin | 33 |
| Haloperidol | 7 |
| Paliperidon | 9 |
| Cariprazin | 1 |
| Risperidon | 10 |
| Quetiapin | 14 |
| Zuclopenthixol | 2 |
| Lurasidon | 2 |
| Flupentixol | 1 |
| Sertindol | 1 |
| Olanzapin | 9 |

**Supplementary Table 6**. Antipsychotic medication and the number of patients taking it in our study

Supplementary Figures


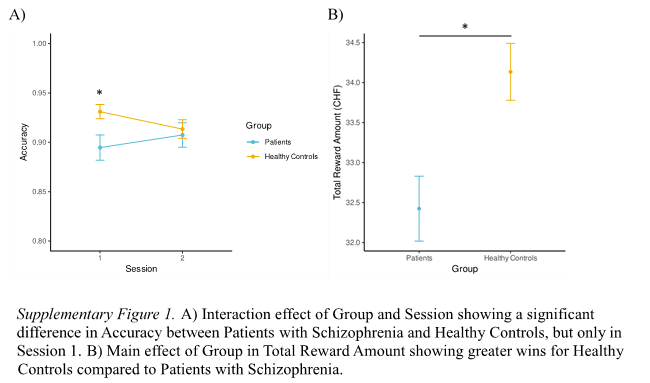


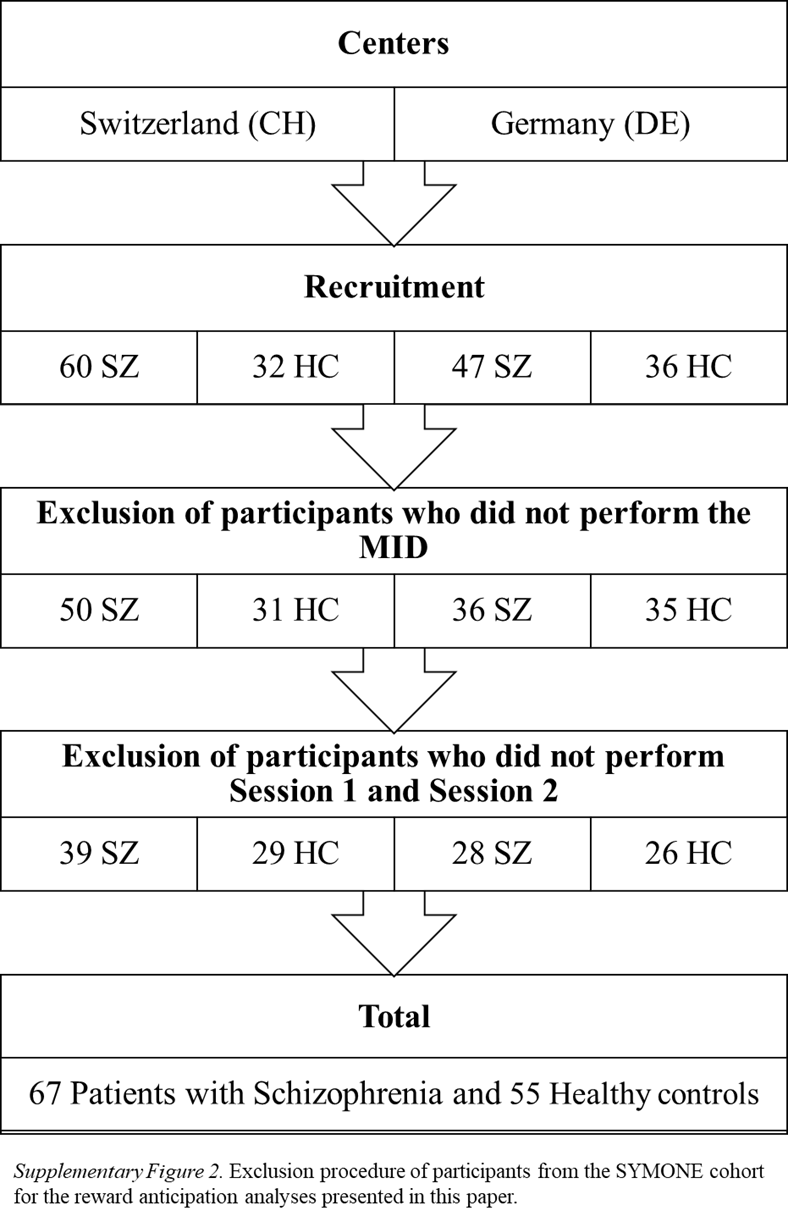


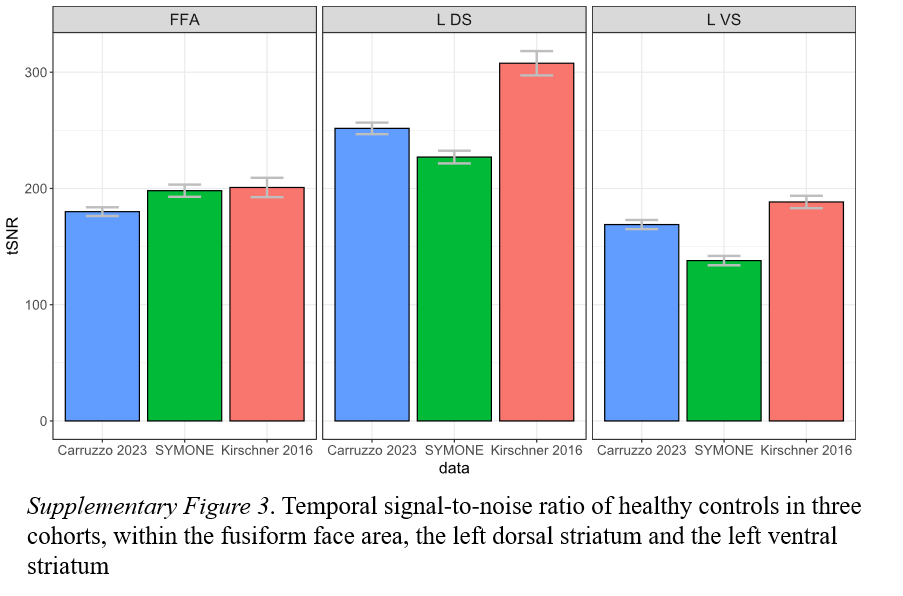


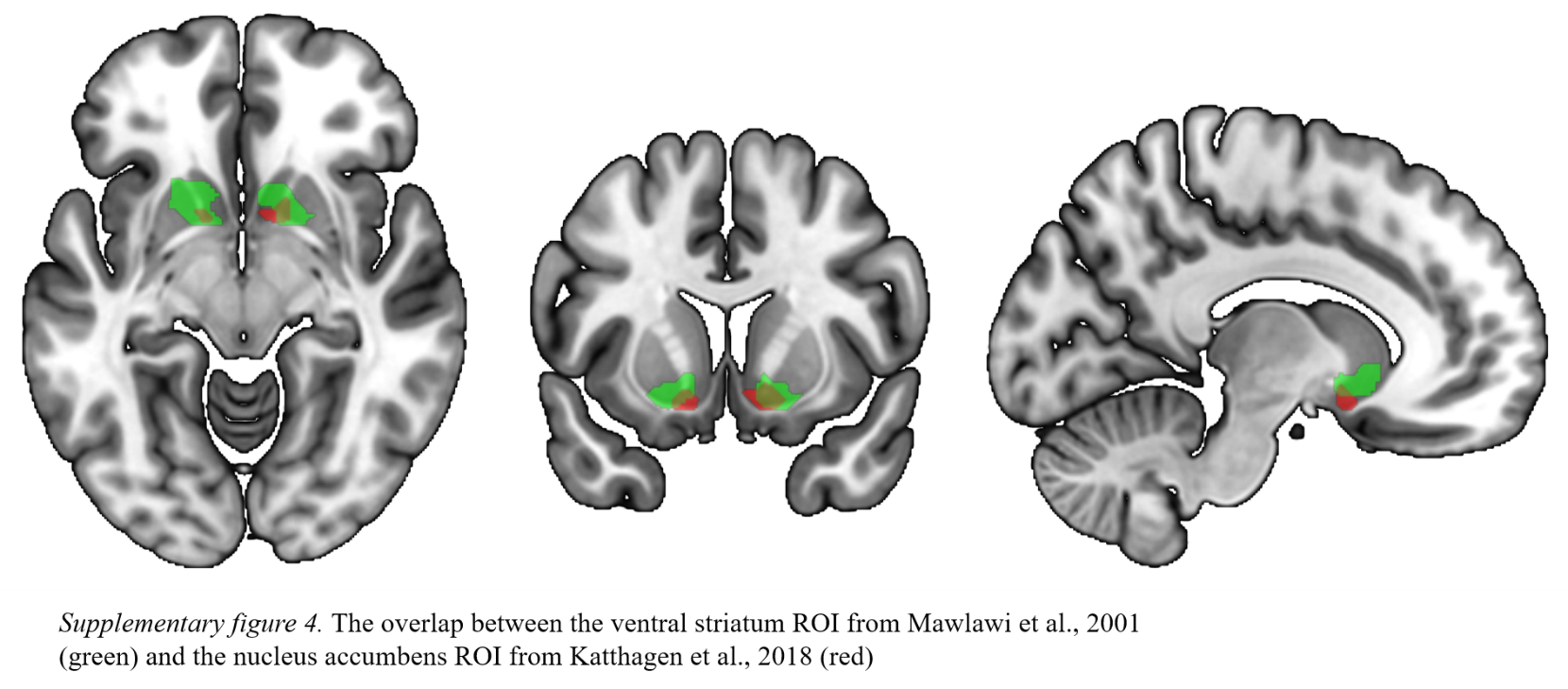

Supplement: sbae046_suppl_Supplementary_Materials [file sbae046_suppl_supplementary_materials.docx]
